# The 1,3‐Dipolar Cycloaddition: From Conception to Quantum Chemical Design

**DOI:** 10.1002/asia.202200553

**Published:** 2022-07-28

**Authors:** Steven E. Beutick, Pascal Vermeeren, Trevor A. Hamlin

**Affiliations:** ^1^ Department of Theoretical Chemistry Amsterdam Institute of Molecular and Life Sciences (AIMMS) Amsterdam Center for Multiscale Modeling (ACMM) Vrije Universiteit Amsterdam De Boelelaan 1083 1081 HV Amsterdam The Netherlands

**Keywords:** 1,3-Dipolar Cycloaddition, Bioorthogonal Chemistry, Density Functional Calculations, SPAAC, Reactivity

## Abstract

The 1,3‐dipolar cycloaddition (1,3‐DCA) reaction, conceptualized by Rolf Huisgen in 1960, has proven immensely useful in organic, material, and biological chemistry. The uncatalyzed, thermal transformation is generally sluggish and unselective, but the reactivity can be enhanced by means of metal catalysis or by the introduction of either predistortion or electronic tuning of the dipolarophile. These promoted reactions generally go with a much higher reactivity, selectivity, and yields, often at ambient temperatures. The rapid orthogonal reactivity and compatibility with aqueous and physiological conditions positions the 1,3‐DCA as an excellent bioorthogonal reaction. Quantum chemical calculations have been critical for providing an understanding of the physical factors that control the reactivity and selectivity of 1,3‐DCAs. *In silico* derived design principles have proven invaluable for the design of new dipolarophiles with tailored reactivity. This review discusses everything from the conception of the 1,3‐DCA all the way to the state‐of‐the‐art methods and models used for the quantum chemical design of novel (bioorthogonal) reagents.

## Graphical abstract

**1,3‐Dipolar cycloaddition (1,3‐DCA) reactions** are ubiquitous in modern chemistry. In this review, we describe the road from the conception of the 1,3‐DCA all the way to the state‐of‐the‐art methods and models used for the quantum chemical design of novel (bioorthogonal) reagents. Three general approaches towards enhanced 1,3‐DCAs reactivity are discussed, namely, transition metal catalysis, predistortion of the dipolarophile, and the stabilization of the transition state.

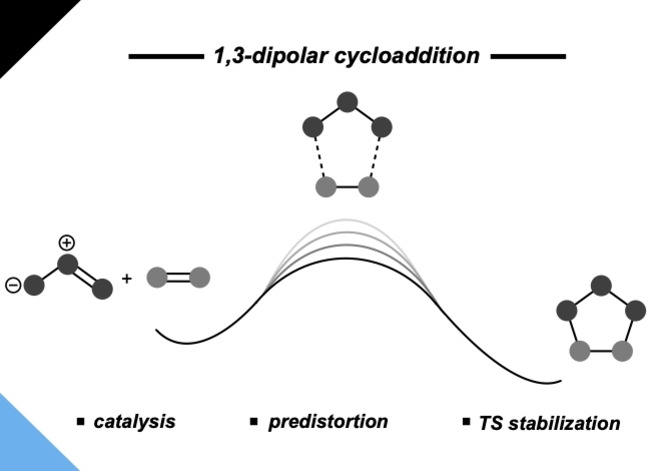

## 1 Introduction

**Scheme 1:**
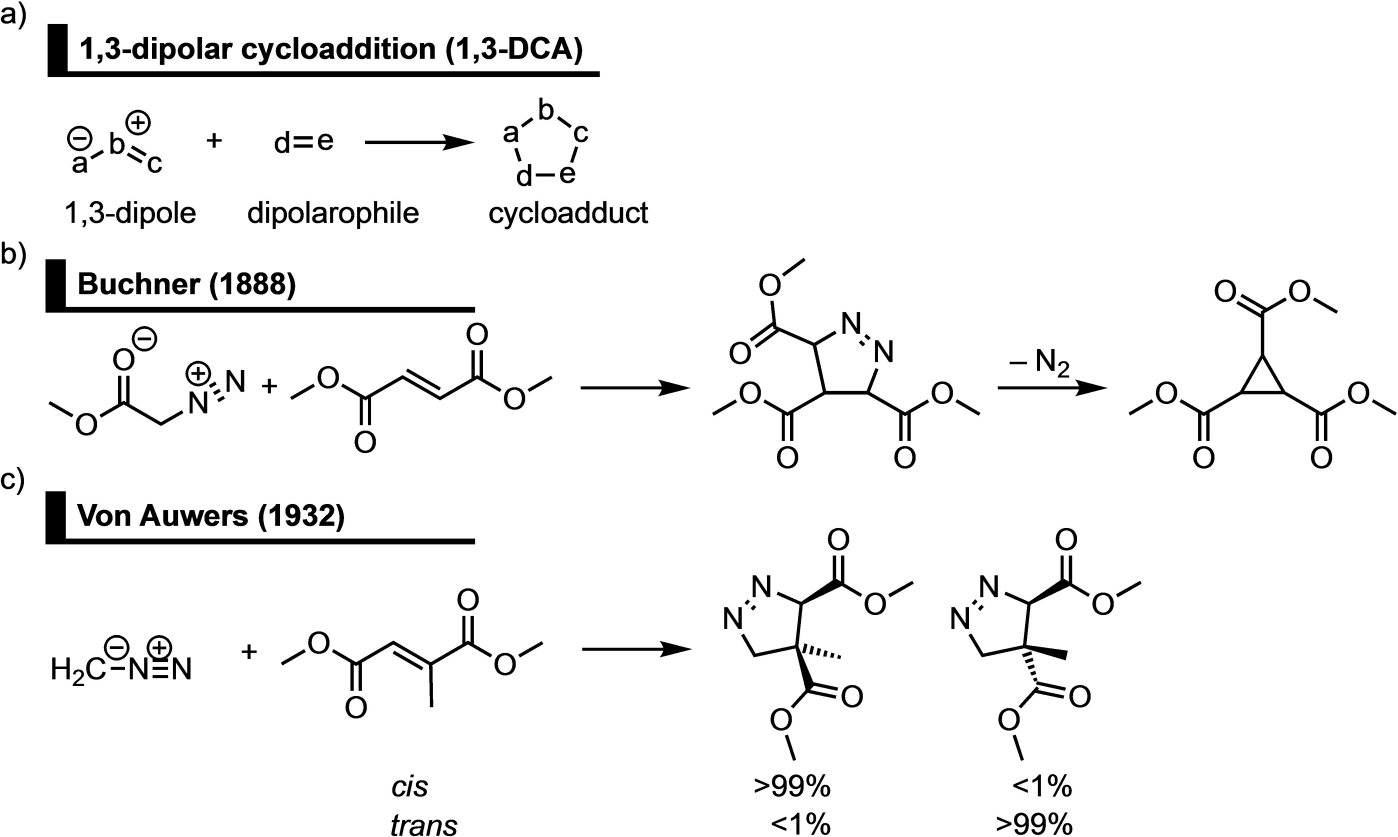
a) Generic 1,3‐dipolar cycloaddition (1,3‐DCA) and the first reported b) 1,3‐DCA^[4]^ and c) stereospecific 1,3‐DCA.^[5]^

The concept of the 1,3‐dipolar cycloaddition (1,3‐DCA), also known as the Huisgen reaction, was first introduced by Rolf Huisgen in 1960.^[1]^ A 1,3‐DCA involves the interaction between a 1,3‐dipole, *i. e*., a dipolar compound with delocalized electrons over three atoms such as azides, and an unsaturated system, the so‐called dipolarophile (Scheme 1).^[1]^ The reaction of the 1,3‐dipole and the dipolarophile furnishes a five‐membered cycloadduct concomitant with the loss of formal charges on the reactants (Scheme 1a). The 1,3‐DCA is quite possibly the most expedient and convenient method to synthesize heterocyclic compounds.^[2]^ Over the years, the 1,3‐DCA has evolved into an iconic organic reaction with applications in various areas of chemistry, ranging from material chemistry to drug discovery.^[3]^

The first 1,3‐DCA between methyl diazoacetate and dimethyl fumaric acid forming trimethyl cyclopropane‐1,2,3‐tricarboxylate was reported in 1888 by the group of Buchner (Scheme 1b).^[4]^ During this reaction the diazoalkane moiety of methyl diazoacetate reacts, as a 1,3‐dipole, with the central C=C double bond of dimethyl fumaric acid, a dipolarophile, yielding a five‐membered cycloadduct, which gives trimethyl cyclopropane‐1,2,3‐tricarboxylate after expulsion of N_2_. The first stereospecific 1,3‐DCA, on the other hand, was reported in 1932 by the group of Von Auwers, who obtained two distinct cycloadducts upon the reaction of diazomethane with *cis*/*trans*‐isomeric unsaturated carboxylic acids. They found that the *cis*‐isomeric unsaturated carboxylic acid exclusively forms the *cis*‐cycloadduct, whereas the *trans*‐isomeric unsaturated carboxylic acid solely yields the *trans*‐cycloadduct (Scheme 1c).^[5]^

**Scheme 2:**
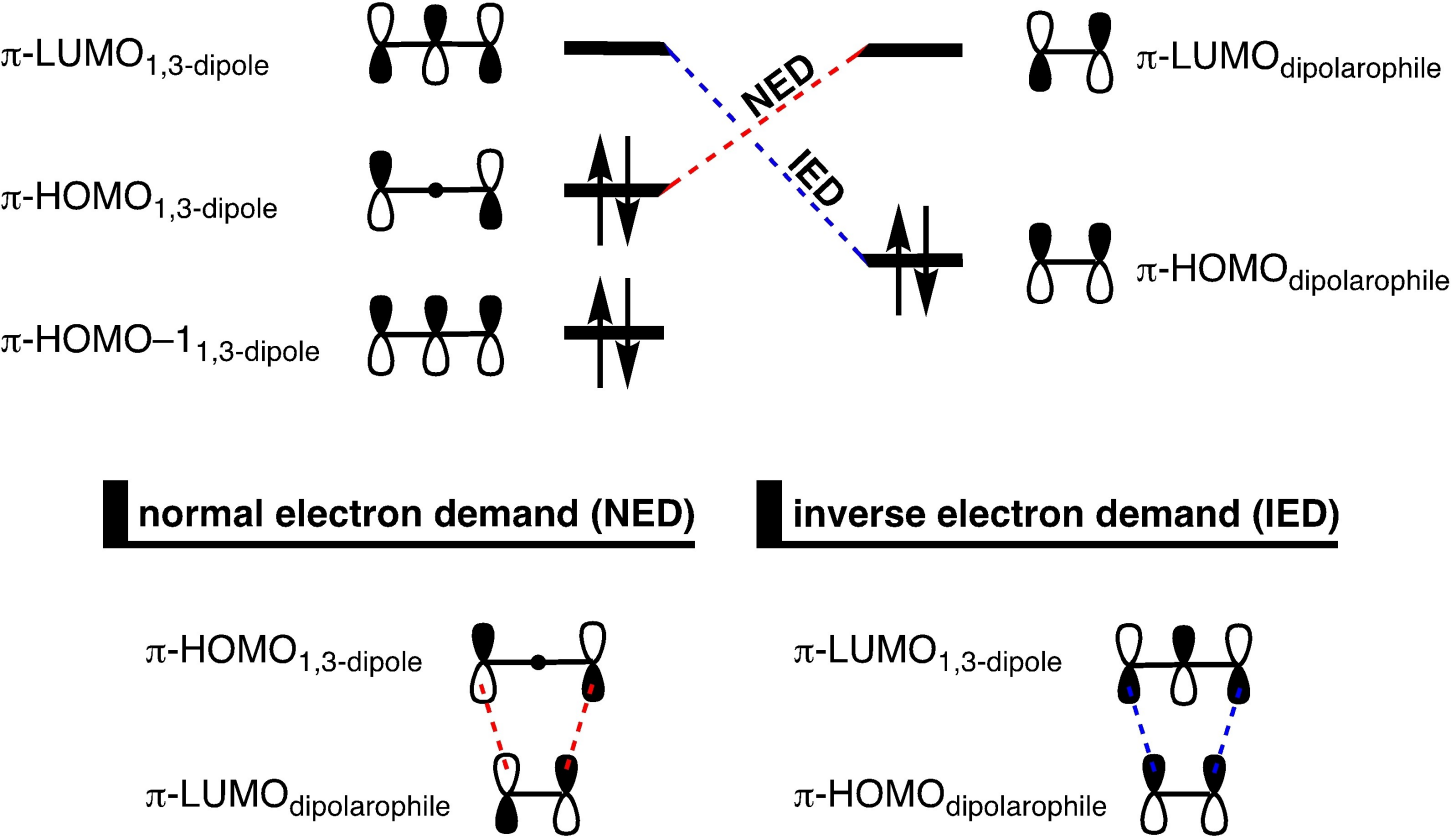
Classification of 1,3‐DCA based on the dominant interaction in the FMO orbitals between the 1,3‐dipole and dipolarophile.

After this, a robust and intuitive framework to rationalize the reactivity of 1,3‐DCAs was needed to predict and rationalize the interactions between the 1,3‐dipole and dipolarophile. Fukui and coworkers proposed to use the energy and shape of the frontier molecular orbitals (FMOs), that is, the highest occupied molecular orbital (HOMO) and the lowest unoccupied molecular orbital (LUMO), of the reactants.^[6]^ In 1971, Sustmann applied FMO theory to describe the reactivity of phenyl azide with a set of dipolarophiles.^[7]^ There, a correlation was observed between the second‐order rate constants and the experimentally determined ionization potentials of the dipolarophiles. Koopmans’ theorem states that, in Hartree–Fock (HF) theory, the ionization potential is equal to the negative orbital energy of the HOMO and hence provides, in practice, an approximate measure of that FMO energy.^[8]^ This correlation was confirmed by the group of Houk, where the experimental findings of Sustmann were successfully reconstructed by calculating the ionization energies using a combination of HF and density functional theory (DFT).^[9]^ Based on the observed correlations, FMO theory permits the classification of 1,3‐DCAs into three classes, which describes the relative energies of the HOMOs and LUMOs on both reactants: i) normal electron demand (NED), HOMO_1,3‐dipole_–LUMO_dipolarophile_; ii) inverse electron demand (IED), LUMO_1,3‐dipole_–HOMO_dipolarophile_, and iii) combination of both (Scheme 2). By classifying the reaction type, both the reactivities and regioselectivities of different cycloadditions can be partially rationalized.

Remarkable progress has been made towards developing a physical understanding of the factors that control the reactivity and selectivity of 1,3‐DCAs and, naturally, this insight can be directly used in the design of novel reactions with specialized applications. In this review, we aim to summarize the most important developments made towards the active tuning and design of 1,3‐DCAs using quantum chemical calculations. A special focus is placed on the design of 1,3‐DCAs with tailored reactivities in bioorthogonal reactions.

## 2 1,3‐Dipolar Cycloaddition

**Scheme 3:**
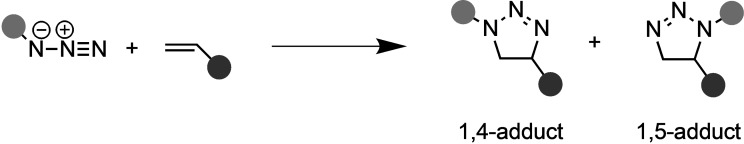
Formation of 1,4‐ and 1,5‐cycloadducts via a 1,3‐DCA.

Useful physical models to rationalize the trends in reactivity of 1,3‐DCA reactions are usually curated by first quantifying the kinetics and thermodynamics for electronically diverse substrates with electron‐donating and electron‐withdrawing substituents. Vilhena *et* *al*. computationally investigated the 1,3‐DCA reaction of hydrozoic acid with various substituted alkenes (R−HC=CH_2_, where R=CH_3_, OH, OCH_3_, NH_2_, CN, and NO_2_) at ωB97X‐D/6‐311++G(d,p).^[10]^ Three main reactivity trends were observed. Firstly, dipolarophiles with electron‐donating substituents exhibited a lower activation barrier for the 1,5‐cycloaddition than for the 1,4‐cycloaddition, whereas electron‐withdrawing substituents switched the preference towards the 1,4‐cycloaddition (Scheme 3). Secondly, the *endo* pathway was slightly preferred over the *exo* pathway. Thirdly, the activation barrier for the 1,3‐DCA towards the 1,5‐cycloadduct decreased upon increasing the electron‐donating capability of the substituent of the dipolarophile from NH_2_ to OCH_3_ to CH_3_ to OH. Similarly, the 1,3‐DCA activation barrier towards the 1,4‐cycloadduct decreased when the electron‐withdrawing capacity of the substituents increased from CN to NO_2_. This reversal in reactivity trends was readily rationalized by analyzing the FMOs. The HOMO_dipolarophile_ was consistently more destabilized when the substituents increase in electron‐donating capacity, which resulted in a favorable decrease of the IED LUMO_1,3‐dipole_–HOMO_dipolarophile_ orbital energy gap. Conversely, stronger electron‐withdrawing substituents on the dipolarophile caused a significant stabilization of the LUMO_dipolarophile_, yielding a smaller, and hence more favorable, NED HOMO_1,3‐dipole_–LUMO_dipolarophile_ orbital energy gap. This study showed the validity of the FMO approach in predicting, and understanding, reactivity trends for substituted dipolarophiles for 1,3‐DCAs.

Ess *et* *al*. studied the activation and reaction energies at CBS‐QB3 for the 1,3‐DCA reaction between two dipolarophiles, namely, ethylene and acetylene, and nine different 1,3‐dipoles based on diazonium betaines, nitrilium betaines, and azomethine betaines (Z^−^−N^+^≡N, Z^−^−N^+^≡CH, Z^−^−NH^+^≡CH_2_, respectively, where Z=O, NH, CH_2_).^[11]^ Changing the 1,3‐dipole from oxides to imines to ylides, a decrease in activation barrier height was observed along this series with a ΔΔ*E* ^≠^ of 6 kcal mol^−1^. The 1,3‐DCAs between nitrilium ylide and ethylene and acetylene were outliers, as the activation energy was about 6 kcal mol^−1^ too high to follow this trend in activation barriers. The activation barriers for the 1,3‐DCA of ethylene were very similar to those of acetylene, with differences of no more than 1.5 kcal mol^−1^. This finding was, however, unexpected, due to the considerable difference in FMO energy gaps and very different reaction energies for these two dipolarophiles. The authors noted that, for the 1,3‐dipole series (Z=O, NH, CH_2_), the lowering of the activation barrier is concomitant with a change from a late to an early transition state. To investigate the origin of the trends in activation barriers, the activation energies at B3LYP/6‐31G(d) were analyzed by means of the activation strain model (ASM)^[12]^ of reactivity, also known as the distortion/interaction model.^[11]^ This analysis method involved decomposing the total electronic energy, for example, the activation barrier, into two terms: the strain energy (Δ*E*_strain_) that results from the distortion of the individual reactants and the interaction energy (Δ*E*_int_) accounting for all mutual interactions between the 1,3‐dipole and dipolarophile. The activation barrier and strain energy of the transition state structure were shown to have a linear correlation, which was striking as the strain energy is not accounted for in the qualitative FMO method. The strain energy of the transition state structure originated predominately from bending the internal angle of the 1,3‐dipole from a near‐linear geometry towards a bent cycloadduct‐like structure, to accommodate for both a small FMO energy gap as well as a good orbital overlap with the dipolarophile. Therefore, the strain energy was related to the stability of the 1,3‐dipole, which had a more stabilized HOMO with a more electronegative atom Z. Thus, the reactivity trends for all 1,3‐dipole series with varying atom Z were determined by the activation strain needed to obtain the geometry where the HOMO–LUMO gap is sufficiently small.

**Figure 1:**
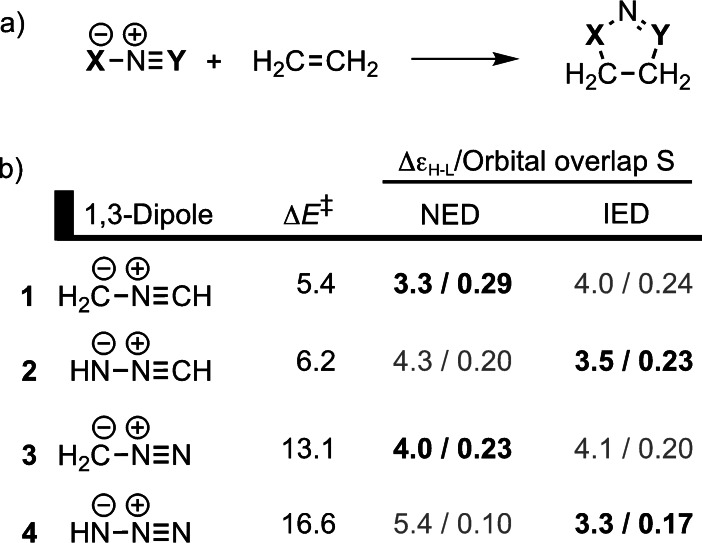
Activation barriers (in kcal mol^−1^), normal electron demand (NED) and inverse electron demand (IED) HOMO–LUMO energy gaps (in eV), and NED and IED HOMO–LUMO orbital overlaps of the 1,3‐DCA reaction between different 1,3‐dipoles and ethylene with the most significant orbital interactions in boldface.^[13]^

Hamlin and coworkers showed that performing the ASM solely on the transition state structures, rather than along the entire reaction pathway, provides an incomplete picture of the factors controlling the 1,3‐DCA reactivity.^[13]^ They demonstrated that the difference in the activation barrier is, in contrast to the prior discussed work by Ess *et* *al*., not strain‐controlled, but, in fact, orbital interaction‐controlled. After a benchmark study using the QMflow program,^[14]^ which facilitates automated workflows of quantum chemical calculations, seven archetypal aza‐1,3‐DCAs were investigated at BP86/TZ2P using the ASM in conjunction with the energy decomposition analysis (EDA).^[15]^ This canonical EDA decomposed the interaction energy (Δ*E*_int_) between the 1,3‐dipole and dipolarophile, obtained from the ASM, into three physically meaningful terms: the classical electrostatic interaction (Δ*V*_elstat_), the Pauli repulsion (Δ*E*_Pauli_) between occupied closed‐shell orbitals on both reactants, and the stabilizing orbital interactions (Δ*E*_oi_) that account, amongst other, for HOMO–LUMO interaction. The two series of linear and bent aza‐1,3‐dipoles comprised: (i) nitrile ylide (**1**), nitrile imine (**2**), diazomethane (**3**), and hydrazoic acid (**4**); and (ii) azomethine ylide (**5**), azomethine imine (**6**), and azonium imine (**7**). Activation strain analyses along the entire reaction coordinate revealed that not the differences in strain energy but, in fact, the differences in interaction energy dictated the trends in reactivity. More specifically, the EDA elucidated that the orbital interactions were the controlling factor behind the reactivity trends of these 1,3‐dipoles. The orbital interactions became less stabilizing as the number of heteroatoms in the 1,3‐dipole increased from 1,3‐dipole **1** to **4** and **5** to **7**. Quantitative Kohn‐Sham molecular orbital analyses revealed the importance of both the NED HOMO_1,3‐dipole_–LUMO_dipolarophile_ and IED LUMO_1,3‐dipole_–HOMO_dipolarophile_ orbital interactions. However, it was the stabilization of the HOMO_1,3‐dipole_, upon increasing the number of heteroatoms in the 1,3‐dipole, that increased the NED orbital energy gap and, consequently, raised the activation barrier. The energies of the HOMO_1,3‐dipole_ and LUMO_1,3‐dipole_ orbitals decrease as the number of nitrogen atoms increases, due to the more electronegative nature of nitrogen compared to carbon. This leads to an increase in the NED HOMO_1,3‐dipole_–LUMO_dipolarophile_ orbital energy gap and a decrease in the IED LUMO_1,3‐dipole_–HOMO_dipolarophile_ orbital energy gap. Additionally, the orbital overlap decreased as the number of nitrogen atoms increased, which can be explained by the contracted nature of the 2*p* atomic orbital of nitrogen compared to carbon.^[16]^ As seen in Figure 1, dipoles **1** and **3** prefer the NED interactions and **2** and **4** prefer the IED interactions.^[13]^ Overall, the activation barrier increases as the number of nitrogen atoms increase, due to less favorable orbital interactions, governed by the decreasing orbital overlap and by the differences in the HOMO–LUMO orbital energy gap of the reactants.

**Figure 2:**
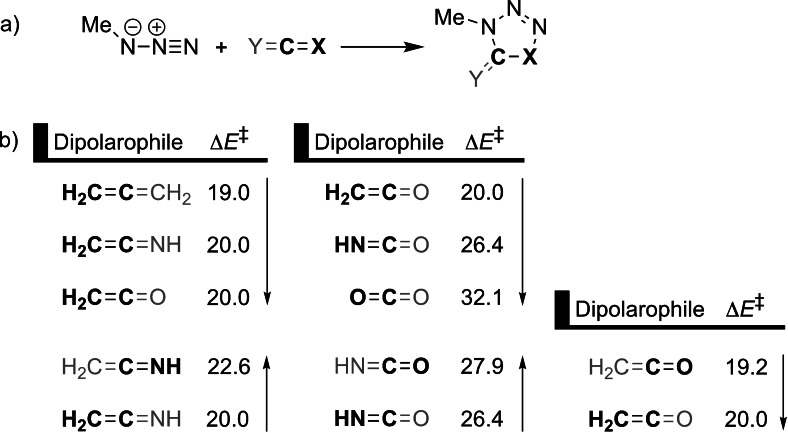
Activation barrier (in kcal mol^−1^) of the 1,3‐DCA reaction between methyl azide and various heteroallenes, where the side of the dipolarophile involved in the 1,3‐DCA are highlighted in boldface and the arrow shows the direction of increased activation barrier.^[17]^

Sometime later, Hamlin and coworkers investigated the effect of heteroatom substitution in the dipolarophile on the 1,3‐DCA reactivity. They computed the activation barriers for the reaction between methyl azide and various linear (hetero)allenes (X=C=Y; X, Y=CH_2_, NH, O; attack at the X=C side) (Figure 2).^[17]^ Three primary reactivity trends emerged from this study. First, the activation barrier increased as Y became more electronegative going from Y=CH_2_ to NH to O, which could be attributed to the more rigid backbone of the dipolarophile with a larger electronegativity difference, due to increased C=Y bond strength.^[18]^ This resulted in increased strain energy during the reaction, since it is more difficult to deform a stronger C=Y bond, and thus an increased activation barrier. Because Y does not directly participate in the 1,3‐DCA reaction, the interaction energy did not change significantly and had, therefore, no impact on the increased activation barrier.

Second, the activation barrier increased more prominently when X of X=C=O became more electronegative, going from 20.0 to 26.4 to 32.1 kcal mol^−1^ for X=CH_2_, NH, and O. Due to the increased electronegativity from X=CH_2_ to NH to O, both the HOMO_dipolarophile_ and LUMO_dipolarophile_ of the heteroallene were stabilized. Accordingly, the LUMO_1,3‐dipole_–HOMO_dipolarophile_ gap was increased and the IED interaction was less stabilizing. In addition, the orbital overlap between the reactants decreased, due to the contracted nature of the 2*p* atomic orbital of the more electronegative X atom. This weakened the favorable orbital interactions and, therefore, resulted in an increased activation barrier.

Third, for the asymmetric heteroallenes, the 1,3‐dipole could attack at two different sides, namely, the X=C and C=Y. However, it had been shown that the 1,3‐dipole preferably attacks the least electronegative of the two terminal atoms, the exception being the allene **CCO**, where attack at the CO side is slightly more favorable than the CC side (*vide infra*).^[17]^ The activation strain model in combination with Kohn‐Sham molecular orbital theory showed that there was a significantly more favorable IED interaction for the attack at the side that consists of the less electronegative atom. This could, in turn, be attributed to more diffuse and energetically less stable HOMO_dipolarophile_ located on the less electronegative atom of the heteroallene. In the case of **CCO**, the strain energy for the attack at the CC side is more destabilizing than the analogous attack at the CO side. This increased strain energy originates from the terminal carbon atom, which needs to pyrimidalize from its trigonal planar equilibrium geometry to a tetrahedral geometry during the 1,3‐DCA reaction, overcoming the more favorable interaction energy. The activation barrier is, therefore, higher for the attack at the CC side and hence steers the preference of attack to the CO side.

## 3 Metal‐Catalyzed Azide‐Alkyne Cycloaddition

The Huisgen reaction was already a well‐established method for the synthesis of heterocycles before the concept of click chemistry was introduced, of which the copper‐catalyzed azide‐alkyne cycloaddition (CuAAC) is arguably the most elementary reaction.^[19]^ Tornøe and Meldal first reported the CuAAC reaction in 2001, but it was not until the work of Meldal and Sharpless in 2002, which highlighted the unprecedented selectivity, robust nature, and scope, that this reaction garnered the spotlight.[^2^, ^20^] The CuAAC reaction proceeds under mild conditions with high yields, regioselectivities, and is tolerant to aqueous environments. In contrast to the thermal (uncatalyzed) reaction, the copper‐catalyzed reaction converts organic azides and terminal alkynes exclusively to the 1,4‐disubstituted 1,2,3‐triazoles as a result of the catalytic mechanism that involves multiple organocopper intermediates (*vide infra*). Because of these advantages (*vide supra*), the CuAAC reaction has found applications in a multitude of fields including organic synthesis, chemical biology, and material sciences.^[21]^

**Scheme 4:**
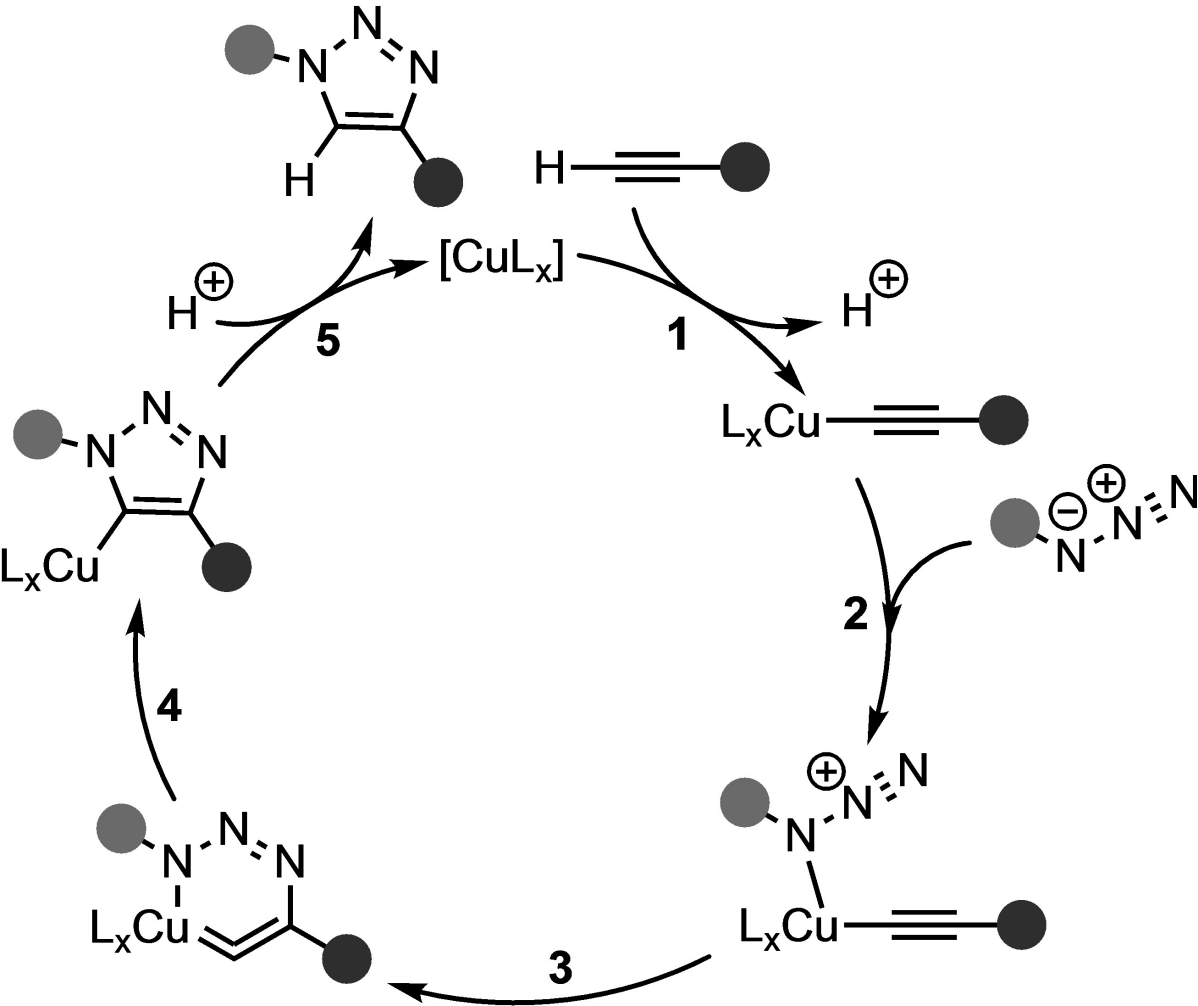
Proposed reaction pathway of mononuclear copper(I) acetylides with organic azides, based on DFT calculations.[^22^, ^26a^]

Theoretical investigations of the CuAAC reaction have primarily been aimed at elucidating the operative catalytic mechanism. Initial computational studies focused on the possible reaction pathways available between mononuclear copper(I) acetylides and organic azides (Scheme 4).^[22]^ It was proposed that the formation of the copper(I) acetylide (**1**; Scheme 4) is followed by the activation of the azide by coordination to the copper center (**2**; Scheme 4). Next, the first C−N bond is formed (**3**; Scheme 4), which is the rate‐determining step of the catalytic cycle, resulting in a strained six‐membered copper metallacycle, formally altering the oxidation state of the copper center from +1 to +3.^[23]^ The activation barrier for this bond formation process was significantly lower than for the analogous reaction step of the uncatalyzed reaction, that is, 18.7 kcal mol^−1^ for the CuAAC reaction compared to 26.0 kcal mol^−1^ for the uncatalyzed analog, exemplifying the observed rate acceleration accomplished by copper catalysis. The formation of copper triazolide (**4**; Scheme 4) was energetically favorable and reduced the copper center from an oxidation state of +3 to +1. If sufficiently bulky, the copper triazole could be isolated.^[24a]^ At last, the copper triazole can be protonated (**5**; Scheme 4), yielding the triazole cycloadduct and the original copper catalyst. In rare cases of low catalyst loading and high catalytic rates, step **5** (Scheme 4) could be the turnover‐limiting step.^[24]^

**Scheme 5:**
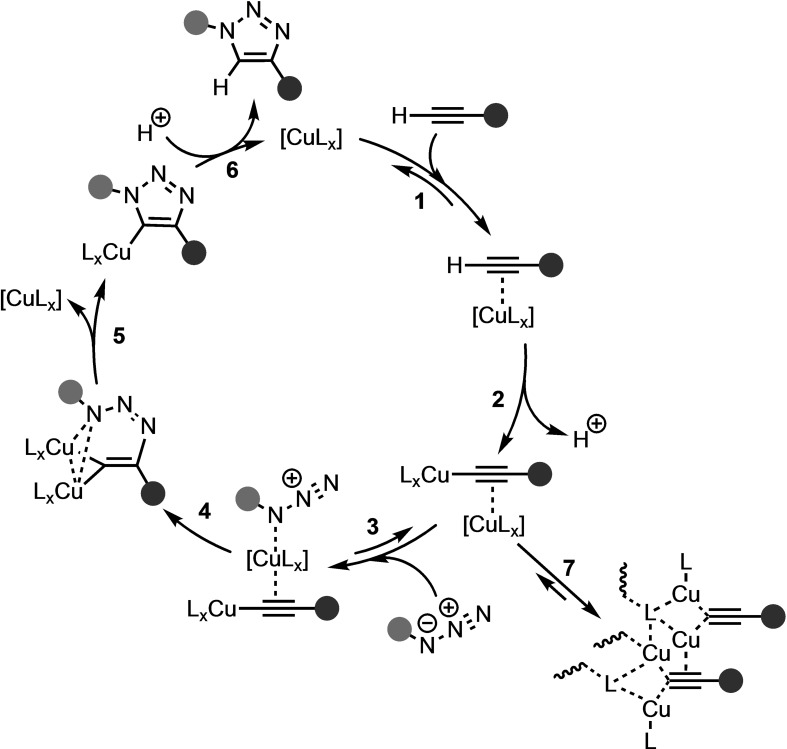
Generally accepted catalytic cycle of the CuAAC reaction, based on DFT calculations, kinetic studies, and experimental results.[^22^, ^25^]

Further investigation of the CuAAC pathway revealed the possibility of the involvement of binuclear copper(I) acetylides.[^22^, ^25^] Kinetic experiments and computational modeling have suggested that the introduction of a second copper center into the metallacycle could efficiently alleviate the ring strain and decrease the activation barrier even further.[^26^, ^27^] The second copper could be introduced during the formation of the copper(I) acetylide, considering the tendency of copper(I) to engage in both σ‐ and π‐bonding with the C≡C triple bond of the acetylene moiety in polymeric and cluster structures (**7**; Scheme 5). A computational study by Straub compared the energetics associated with higher‐order aggregates and the binuclear copper pathways.^[27]^ The Gibbs free energy barriers of the C−N bond formation step for mono‐, di‐, and tetra‐nuclear copper acetylide were 21.2, 18.6, and 20.8 kcal mol^−1^, respectively, at B3LYP/LACV3P++**//B3LYP/LACVP**. Based on these energetic data, the authors concluded that binuclear intermediates were favored over the mono‐ and tetra‐nuclear intermediates.

The group of Fokin sought to provide experimental evidence for the *in silico* derived mechanism.^[22]^ They found that the reaction of 5 mol% copper with phenylacetylene forms higher‐order polynuclear phenyl copper acetylides, which quench the catalytic cycle and should, thus, be avoided. In addition, they observed that the CuAAC did not exhibit uniform second‐order kinetics and furnished evidence against the binuclear copper pathway. Later, Fokin and coworkers confirmed the presence of binuclear copper acetylides in the catalytic cycle through the use of metal isotope crossover methods.^[25]^ In step **2** of Scheme 5, when a σ‐coordinated acetylide was formed, a second copper coordinated to the π‐system that facilitates the 1,3‐DCA. With these experimental findings, the previously proposed CuAAC catalytic cycle was revised (Scheme 5). Recently, Balcells *et* *al*. presented a DFT study of an assymmetric dicopper complex at a PBE0‐GD3/def2SVP and found a concerted cycloaddition step, contrasting the generally accepted two step mechanism (**4** and **5**; Scheme 5).^[28]^ They ascribed the concerted cycloaddition step to the high saturation of the two copper centers.

The copper catalyst of the CuAAC reaction, although the most common, is not the only transition metal able to accelerate the 1,3‐DCA reaction between an azide and an alkyne. High yields and regioselectivity have also been reported for this class of 1,3‐DCA reactions with other transition metal catalysts, but their kinetics, costs, and robustness are, compared to the CuAAC, inferior, especially in the context of click chemistry.

**Scheme 6:**
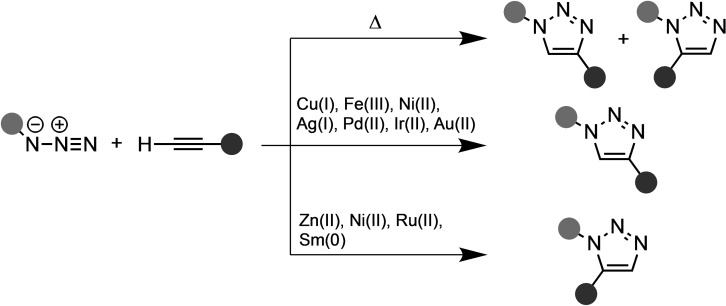
The effect of transition metal catalysts in the regioselectivity of the 1,3‐DCA reaction between an azide and an alkyne.

**Scheme 7:**
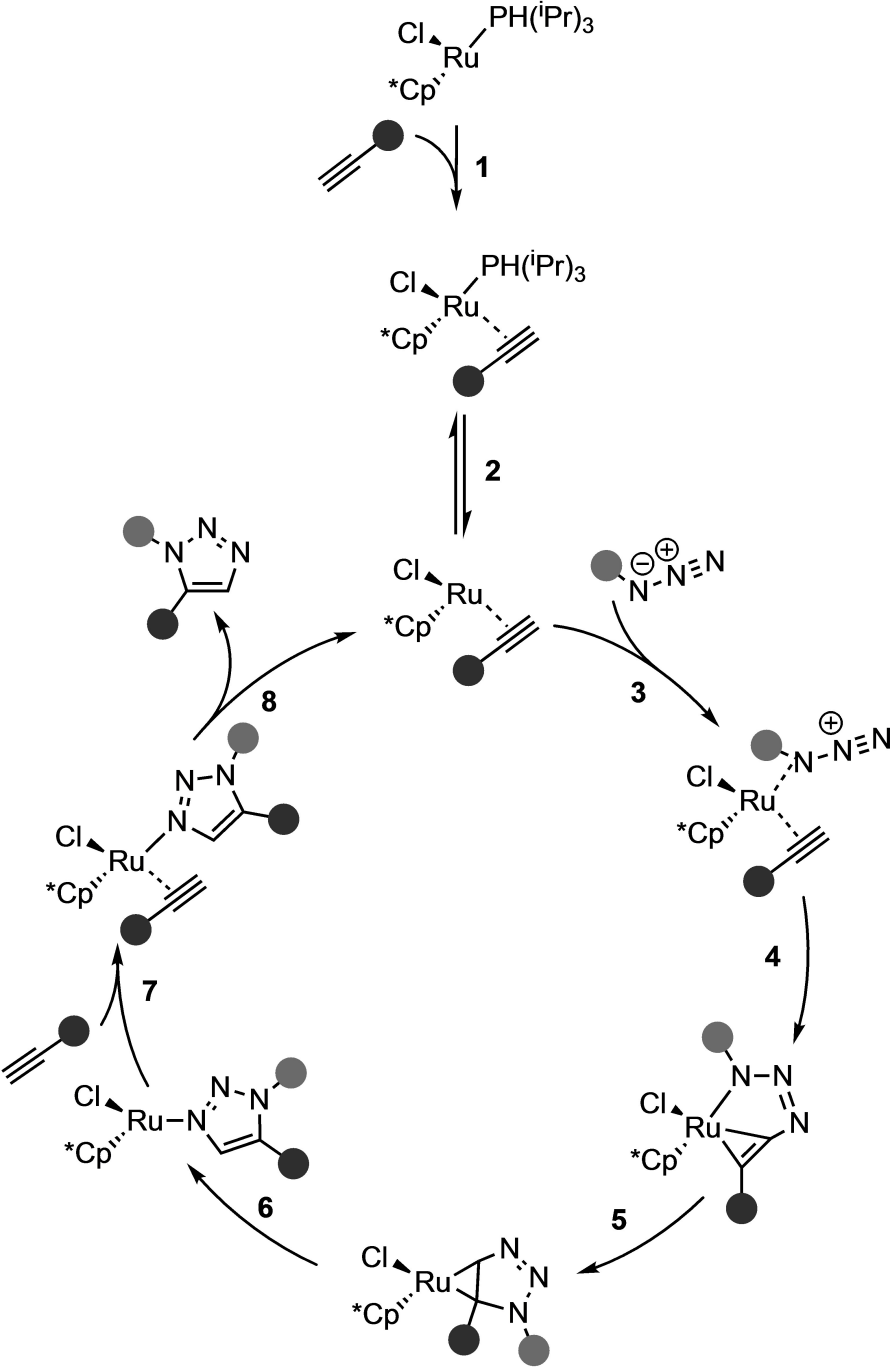
Catalytic cycle of the RuAAC reaction, based on DFT calculations and experimental NMR studies.^[30]^

As mentioned above, the CuAAC is selective for 1,4‐disubstituted 1,2,3‐triazoles under mild conditions with very high yield and regioselectivity. Nevertheless, an efficient catalyst with high regioselectivity towards 1,5‐disubstituted 1,2,3‐triazoles was lacking. Therefore, extensive studies into transition metal‐catalyzed 1,3‐DCAs, using transition metals other than copper, have been performed (Scheme 6). In 2005, Fokin and coworkers presented a novel ruthenium catalyst for the regioselective synthesis of 1,5‐disubstituted and 1,4,5‐trisubstituted 1,2,3‐triazoles from organic azides with terminal and internal alkynes, respectively (Scheme 7).^[29]^ The ruthenium‐catalyzed azide‐alkyne cycloaddition (RuAAC) showed high yields, regioselectivity, and efficiency under mild conditions, but, in contrast to the CuAAC, running the reaction in polar protic solvents (*e. g*., water) gave very low conversion. Nolan and coworkers performed a combined experimental NMR and computational study into the mechanism of the RuAAC reaction.^[30]^ They investigated the Ru(II) catalyst and proposed the overall mechanism shown in Scheme 7. Initially, the alkyne coordinates to the precatalyst, Cp*RuCl(P^i^Pr), to form the experimentally observed Cp*RuCl(η^2^‐HCCPh)(P^i^Pr). DFT calculations indicated phosphine ligand dissociation and subsequent coordination of the organic azide to the ruthenium through the internal nitrogen (**3**; Scheme 7). Next, the terminal nitrogen of the activated azide performs a nucleophilic attack on the terminal carbon of the alkyne (**4**; Scheme 7). This step is almost barrierless with a Δ*G* ^≠^=0.3 kcal mol^−1^. This is followed by C−N oxidative coupling and cyclization (**5**; Scheme 7), forming the metallacyclopropane intermediate, which ultimately isomerizes to the N‐coordinated 1,5‐disubstituted triazole (**6**; Scheme 7). This reaction step had been identified as the rate‐determining step. At last, the new terminal alkyne coordinates to the ruthenium center (**7**; Scheme 7), the triazole was released (**8**; Scheme 7), and the catalyst, Cp*RuCl(η^2^‐HCCPh), is regenerated. The highly exothermic nature of the triazole formation was predicted to be the driving force behind the RuAAC reaction.

It was proposed that the regioselectivity of the CuAAC and RuAAC reactions stems from the coordination of the acetylene to the transition metal. For the CuAAC reaction, a σ‐bonded intermediate is observed (after step **2**; Scheme 5), whereas this coordination mode was ruled out during mechanistic studies of the RuAAC reaction (after step **3**; Scheme 7).^[29c]^ For both the CuAAC and RuAAC, these reaction steps are followed by C−N bond formation, see step **4** in both Scheme 5 and Scheme 7, respectively. During the RuAAC reaction, the C−N bond formation occurs between the more electronegative and less sterically encumbered carbon of the alkyne and the terminal nitrogen of the azide, forming the metallacycle. During the CuAAC reaction, on the other hand, a second copper catalyst engages in σ‐coordination with the acetylene, which facilitates a nucleophilic attack on the terminal nitrogen by the sterically more crowded carbon of the dipolarophile, forming the dinuclear metallacyle.^[25]^ Thus, the coordination mode of the acetylene to the transition metal‐induced the propensity of the regioselectivity towards the 1,4‐ and 1,5 cycloadduct for CuAAC and RuAAC, respectively.

Later, Greany and coworkers found that the addition of a stoichiometric amount of ZnEt_2_, a common Lewis acid, to a solution of a terminal alkyne and an azide dissolved in THF in the presence of catalytic base formed exclusively the 1,5‐cycloadduct.^[31]^ Additionally, Hong *et* *al*. investigated several lanthanides as catalysts and found that a samarium complex, Sm[N(SiMe_3_)_2_]_3_ (5 mol%), with n‐BuNH_2_ (10 mol%) in toluene at 50 °C showed high regioselectivity towards the 1,5‐disubstituted cycloadduct.^[32]^ The authors proposed a reasonable mechanism, but no deeper investigation was presented. Kim *et* *al*. presented a nickel‐catalyzed azide‐alkyne cycloaddition to furnish 1,5‐disubstituted 1,2,3‐triazoles from readily available substrates and inexpensive reagents at room temperature.^[33]^ This NiAAC reaction is a potent candidate for click chemistry as it is highly compatible with water as solvent and shows excellent regioselectivity. More recently, Cossío and coworkers introduced highly enantio‐ and diastereoselective 1,3‐DCAs that were catalyzed by silver and copper complexes.^[34]^

Since the introduction of CuAAC, the reaction has played a prominent role, especially in the context of click chemistry, because of its exceptional reactivity, orthogonality, and regiospecificity towards the 1,4‐disubstituted 1,2,3‐triazole. Ruthenium catalysts have been shown to catalyze the azide‐alkyne 1,3‐DCA reaction towards the 1,5‐disubstituted cycloadduct with high regiospecificity and tolerance to a wide range of substituents under mild conditions.^[29]^ The initial activation of the alkyne, as a consequence of the increased nucleophilicity of the π‐system through backdonation from the transition metal, was proposed to be identical in CuAAC and RuAAC. The subsequent coordination mode of the alkyne to the transition metals was suggested to cause the regiospecificity towards the 1,4‐cycloadduct and 1,5‐cycloadduct, respectively. Both reactions exhibit high reactivities and regioselectivities at ambient temperatures and hence are effective candidates for the synthesis of novel heterocyclic compounds.

## 4 Strain‐Promoted Azide‐Alkyne Cycloaddition

**Scheme 8:**
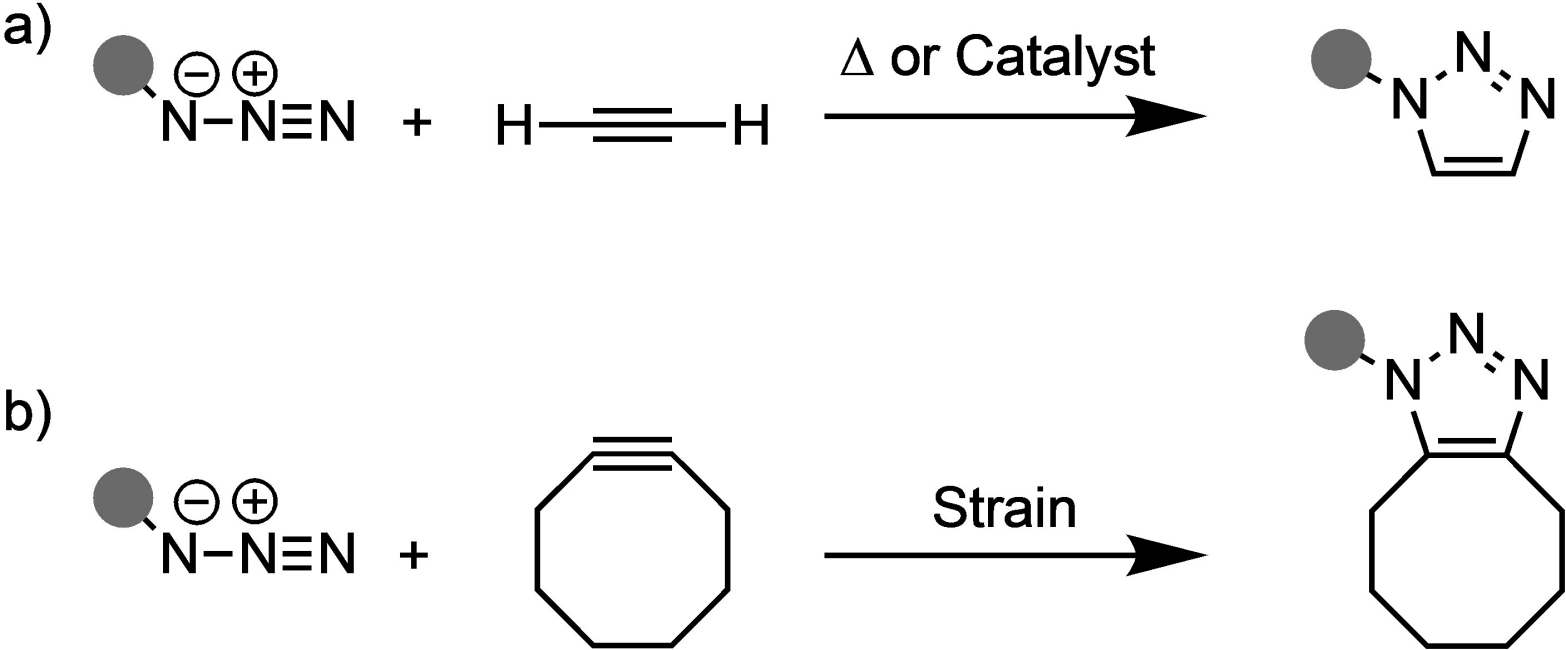
The reaction rates for azide‐alkyne 1,3‐DCA reaction can be increased by a) elevated temperatures, a catalyst, or b) the inclusion of strain in the alkyne dipolarophile.

The first examples of 1,3‐DCAs between azides and strained cyclooctynes by Wittig *et* *al*. and Blomquist *et* *al*. were shown to proceed with high selectivity and reactivity without the need for a catalyst (Scheme 8).^[35]^ It was originally proposed that the strained nature of the cycloalkynes, which nicely resembled the geometry it needed to adopt in the transition state, was the reason for the enhanced reactivity. Due to their excellent selectivities and reactivities at ambient temperatures, these so‐called strain‐promoted azide‐alkyne cycloaddition (SPAAC) reactions were adopted by Bertozzi and coworkers as a selective chemical reaction that is bioorthogonal in living systems.^[36]^

**Figure 3:**
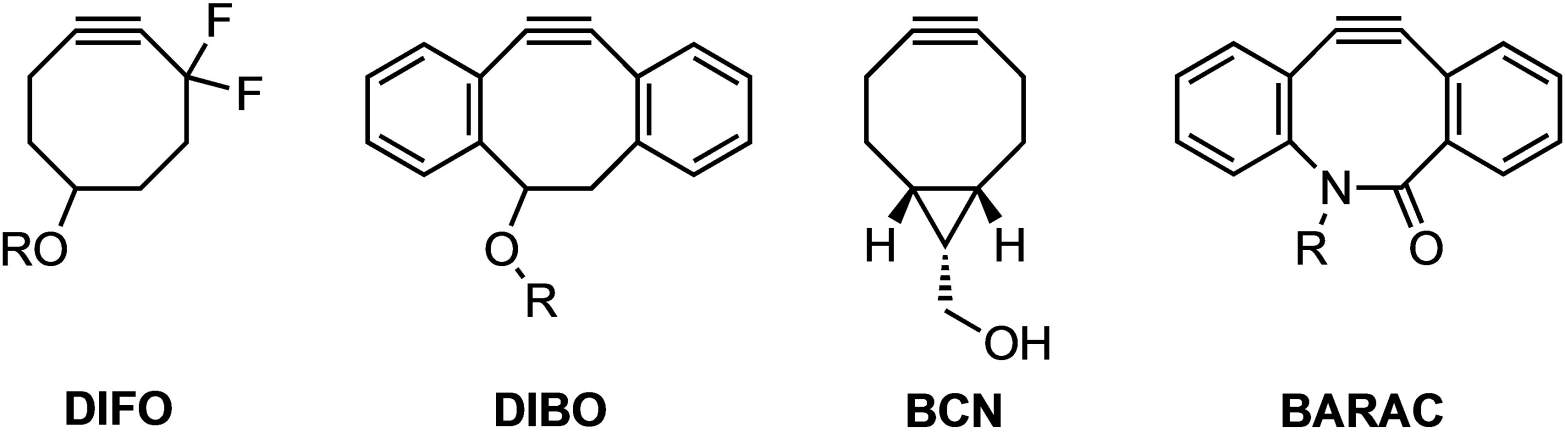
Cycloalkyne reagents that were designed and synthesized with the aim of enhanced reactivity towards 1,3‐DCA with an azide dipole.

The significant role of the SPAAC reaction, especially in the context of bioorthogonal chemistry, has preempted numerous investigations into the role of both strain and electronics on 1,3‐DCA reactivity (Figure 3). Bertozzi and coworkers judicially added electron‐withdrawing substituents on the propargylic position of cyclooctyne and saw a significant enhancement in reactivity.^[37]^ Difluorinated cyclooctyne (DIFO) has also shown to be a reactive bioorthogonal reagent with a second‐order rate constant of 7.6×10^−2^ M^−1^ s^−1^, that is, an order of magnitude faster than previously reported SPAAC reactions involving cyclooctynes. Boons and coworkers took a different approach and showed how increased predistortion by functionalization with aromatic moieties could lead to 4‐dibenzocyclooctynols (DIBO) with second‐order rate constants of 0.17 and 2.3 M^−1^ s^−1^, three times the rate of the parent cyclooctynol, when reacting with benzyl azide in methanol and a mixture of water/acetonitrile (1 : 4 v/v), respectively.^[38]^ The aromatic rings on these reagents can also be functionalized such that they become fluorescent and useful for labeling biomolecules. Van Delft and coworkers increased the strain of the cyclooctyne by addition of a cyclopropane functionality[^39^, ^40^] and developed dicyclo[6.1.0]nonyne (BCN) derivatives that display excellent kinetics and chemoselectivities for azide reagents under physiological conditions. They investigated the reaction kinetics for the SPAAC reaction with benzyl azide and found two orders of magnitude enhancement in reactivity over cyclooctyne.^[40]^ Bertozzi and coworkers combined both electronic and strain‐based design principles in the design of biarylazacyclooctynone (BARAC).^[41]^ BARAC is functionalized with both aromatic moieties, similar to DIBO, and heteroatoms in the cyclooctyne ring that stabilize the transition state through electronic effects. The amide group can participate via resonance between the nitrogen lone pair and the carbonyl group. The second‐order rate constant for the reaction between BARAC and benzyl azide was determined to be 450 times greater than for an unactivated parent cyclooctyne.^[41]^ These examples illustrate the two distinct approaches commonly used in the design of novel cycloalkyne reagents for enhanced SPAAC reactivity, namely, the destabilization of the reactants and the stabilization (*i. e*., lowering) of the transition state.

Remarkably, Dommerholt *et* *al*. found that the SPAAC reaction between electron‐deficient aryl azides and BCN showed enhanced reactivity. This would suggest that the IED LUMO_1,3‐dipole_–HOMO_dipolarophile_ interaction becomes dominant, whereas for SPAAC, in general, the NED HOMO_1,3‐dipole_–LUMO_dipolarophile_ interaction is the dominant orbital interaction.^[42]^ They indeed confirmed, through quantitative Kohn‐Sham molecular orbital analyses, that the dominant donor‐acceptor orbital interaction is the IED interaction between the occupied π‐orbital of BCN and the relatively low‐lying LUMO of the electron‐deficient aryl azide. Later, Xie *et* *al*. revealed that the enhanced reactivity of perfluorinated aryl azides towards benzoannulated cyclooctynes, compared to aryl azide, is likely associated with the two ortho F atoms of the perfluorinated aryl azides, which distort the azide and, thereby, lowering the activation strain of this 1,3‐DCA reaction.^[43]^

**Figure 4:**
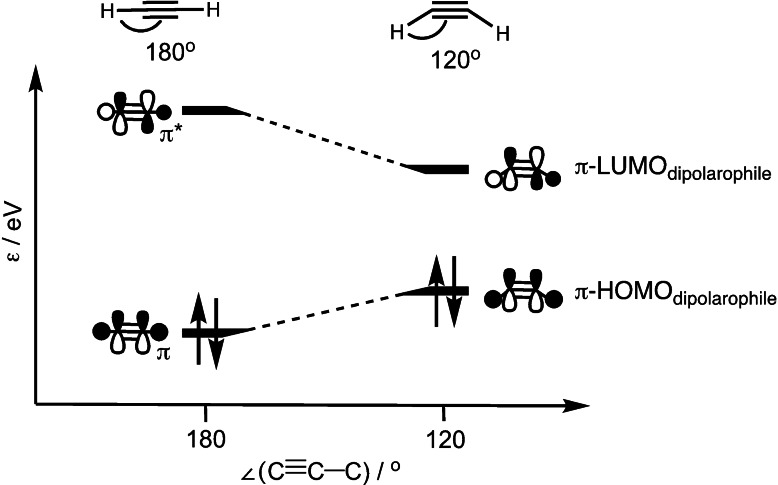
Walsh diagram for the symmetric in‐plane bending of acetylene.^[44]^

The increased reactivity of cycloalkynes in the SPAAC reaction, compared to their linear counterparts, can be traced to the predistortion of the cyclic dipolarophile, which requires less energy to reach the transition state geometry. Hamlin *et* *al*. showed that despite the reduction in activation strain being substantial between linear and cycloalkynes, the differences in SPAAC reactivity of cycloalkynes (*e. g*., seven‐, eight‐ and nine‐membered cycloalkynes) are traced back to their differences in interaction energy with the 1,3‐dipole, in this case, methyl azide.^[44]^ Energy decomposition analyses showed that the gain in orbital interactions caused the increase in stabilizing interaction energy upon decreasing the ring‐size of the cycloalkyne. Through Kohn‐Sham molecular orbital (KS‐MO) analyses, it was found that for both the NED and IED the orbital energy gaps decrease, and the orbital overlaps increase, as the ring size of the cycloalkyne decreased. The decrease in the orbital energy gaps is in line with the destabilization of the π‐HOMO_dipolarophile_ and the stabilization of the π‐LUMO_dipolarophile_ upon in‐plane bending of the alkyne. Upon bending of the C≡C−H moiety of acetylene, the π‐LUMO_dipolarophile_ was stabilized by the in‐phase mixing of its σ*_C−H_ and π*_C≡C_ orbital lobes, while the π‐HOMO_dipolarophile_ was destabilized because of the out‐of‐phase mixing of the σ_C−H_ and π_C≡C_ orbital lobes, effectively reducing the NED and IED orbital energy gaps (Figure 4). Consistent with the findings of Houk and Hoffman, the orbital overlap between the cycloalkyne and azide was enhanced, because the π_C≡C_ and π*_C≡C_ orbitals hybridize, upon C≡C−H bending, in the opposite direction of the σ_C−H_ bond, which gives rise to a larger orbital amplitude at the external face of the π‐HOMO_dipolarophile_ and π‐LUMO_dipolarophile_ pointing towards the azide.^[45]^ These findings demonstrate that both the reduced strain, due to geometric predistortion, and the enhanced orbital interactions play a dominant role in the enhanced reactivity of cyclic alkynes in the SPAAC reaction.

**Figure 5:**
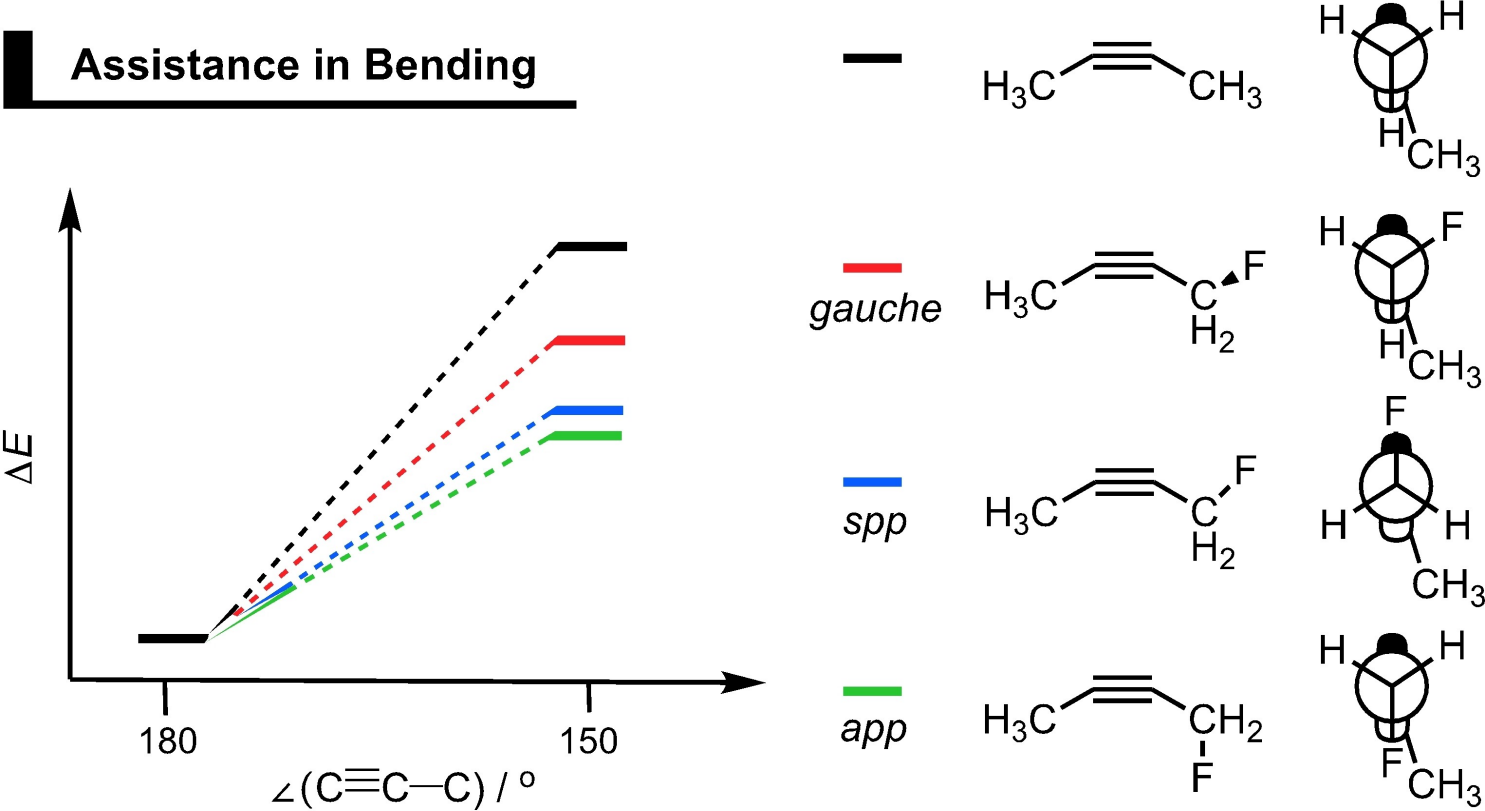
A schematic depiction of the change in relative energy upon symmetric bending of 2‐butyne and the *gauche*, *syn*‐periplanar (*spp*), and *anti‐*periplanar (*app*) arrangements of 1‐fluoro‐2‐butyne shown in the Newman projections that includes the π_in‐plane_ orbital representation.^[46]^

Alabugin and coworkers proposed that fluorine atoms on the propargylic position of the dipolarophile can stabilize the transition state through two distinct stereoelectronic effects, namely, hyperconjugative assistance and C−H⋅⋅⋅F interactions.^[46]^ They performed computations on the 1,3‐DCA reaction between methyl azide and DIFO at a B3LYP/6‐31G(d) level. The authors employed NBO analyses to quantify hyperconjugative effects caused by the donation from the in‐plane alkyne π‐system into the σ*_C−F_ orbital of the C−F bond on the propargylic carbon (π_in‐plane_→σ*_C−F_). In the transition state, these hyperconjugative interactions between the two π‐systems and the σ*_C−F_ increased by 2.8 kcal mol^−1^, resulting in a lowering of the reaction barrier and hence an enhancement of the SPAAC reactivity. To further investigate the stereoelectronic effects, 2‐butyne and 1‐fluoro‐2‐butyne were symmetrically bent at B3LYP/6‐31G(d,p).^[46]^ The dipolarophile reactants’ C≡C−C angles were symmetrically bent to 150°, which is the approximate transition state angle during the SPAAC reaction. The energy penalty associated with this distortion from linear to bent was lowered, relative to the non‐substituted 2‐butyne, by 1.7 kcal mol^−1^ as the σ*_C−F_ acceptor was in the *anti*‐periplanar (*app*; green) arrangement relative to the in‐plane π‐system (Figure 5). The stabilizing effect was 1.6 kcal mol^−1^ and 0.9 kcal mol^−1^ in the *syn‐*periplanar (*spp*; blue) and *gauche* (red) arrangement, respectively. Thus, the bending of the alkyne reactant was assisted by a hyperconjugative interaction (π_in‐plane_→σ*_C−F_), which is enhanced due to the C≡C−C bending. This effect was maximized in the *app* and *spp* arrangements, because the orbital overlap between the collinear π_in‐plane_ and the σ*_C−F_ orbitals are in these arrangements optimized (Figure 5).^[47]^

**Figure 6:**
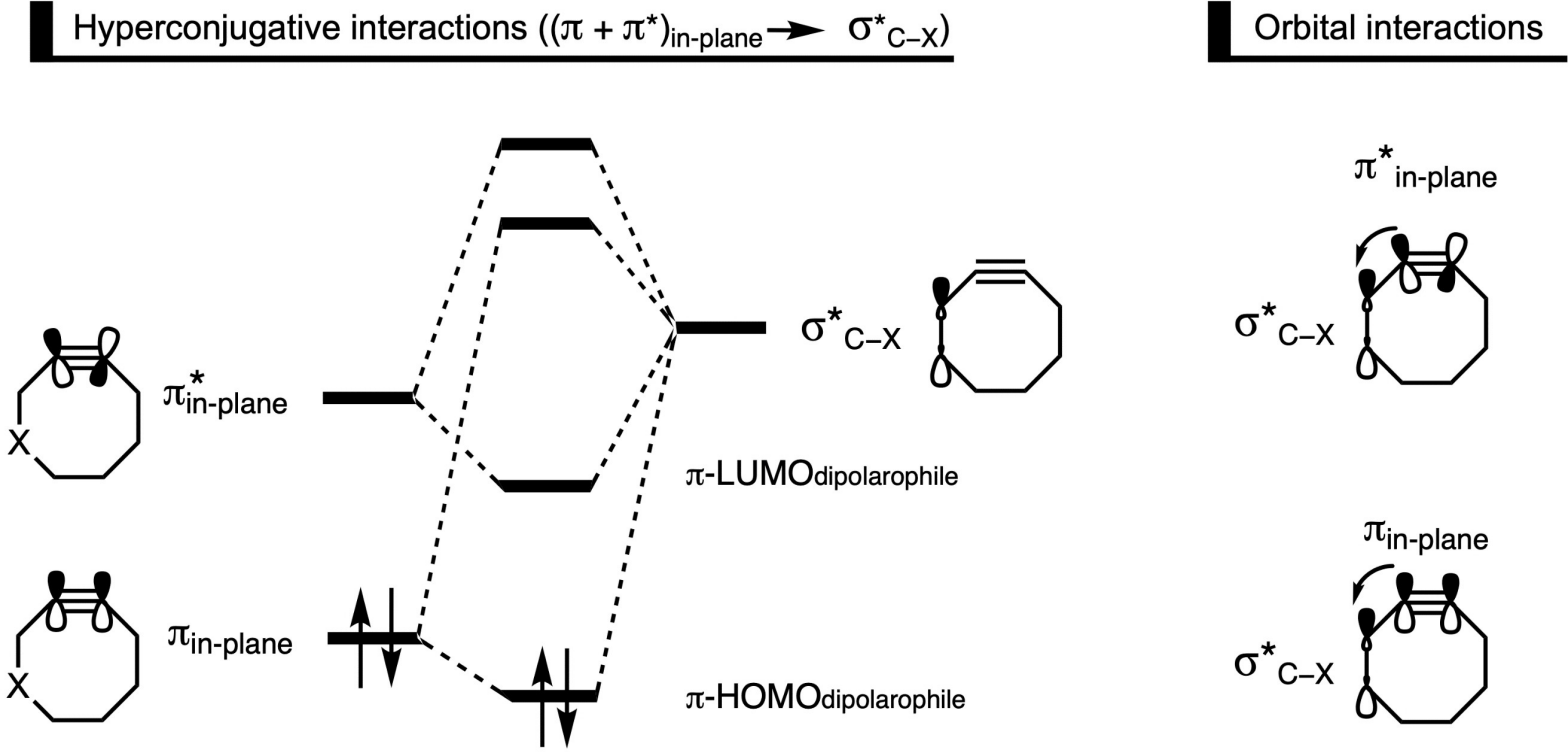
A molecular orbital representation of the hyperconjugative interaction between the in‐plane π‐system and the σ*_C−X_ acceptor (π_in‐plane_→σ*_C−X_ and π*_in‐plane_→σ*_C−X_), which stabilizes the frontier molecular orbitals of the dipolarophile.

The group of Alabugin also investigated the hyperconjugative interactions for the actual transition state of the 1,3‐DCA between methyl azide and 1‐fluoro‐2‐butyne. NBO analyses of the interaction between the in‐plane π‐system into the σ*_C−F_ acceptor orbitals ((π_in‐plane_+π*_in‐plane_)→σ*_C−F_) reveal a substantial increase in the hyperconjugative assistance of the σ*_C−F_ acceptor as the methyl azide approaches the alkyne. Electron density in the π*_in‐plane_, which originates from the 1,3‐dipole during the bond formation process of the 1,3‐DCA reaction, was delocalized into the σ*_C−F_ orbital. NBO analyses showed that the donation from the in‐plane π*‐system into σ*_C−F_ orbital increased compared to the non‐fluorinated 2‐butyne. Again, stereoelectronic effects, observable as the (π_in‐plane_+π*_in‐plane_)→σ*_C−F_ interaction, were highly enhanced in both the 1,5‐*app* and 1,4‐*app* conformers. The increase compared to the isolated alkyne is primarily due to the π*_in‐plane_→σ*_C−F_. Thus, the transition state is stabilized through delocalization of electron density from the π*_in‐plane_ donor, which receives electron density from the HOMO_azide_ during the 1,3‐DCA reaction, to the σ*_C−F_ acceptor orbital (Figure 6).

Two distinct stereoelectronic effects on the reactivity of the 1,3‐DCA between methyl azide and 1‐fluoro‐2‐butyne were observed. At CPCM(H_2_O)‐B3LYP/6‐31G(d), the Gibbs free energy of activation, relative to 2‐butyne, decreased by −2.7, −1.4, −1.8, and −2.4 kcal mol^−1^ for 1,4‐*app*, 1,4‐*gauche*, 1,5‐*app*, and 1,5*‐gauche*, respectively. Aside from the significant stabilization for the *app* conformers, a notable stabilization was observed for the 1,5‐*gauche* transition state. This latter stabilization has been ascribed to the classical electrostatic interaction between the partially positive hydrogen on the methyl group and the partially negative fluoride, *i. e*., C−H⋅⋅⋅F interactions. In all, the authors showed that the fluoride substituent on the propargylic position can reduce the activation barrier via two distinct mechanisms: (i) through hyperconjugative assistance, to both bending (π_in‐plane_→σ*_C−F_) and bond formation (π*_in‐plane_→σ*_C−F_); and (ii) through classical electrostatic C−H⋅⋅⋅F interactions.

**Figure 7:**
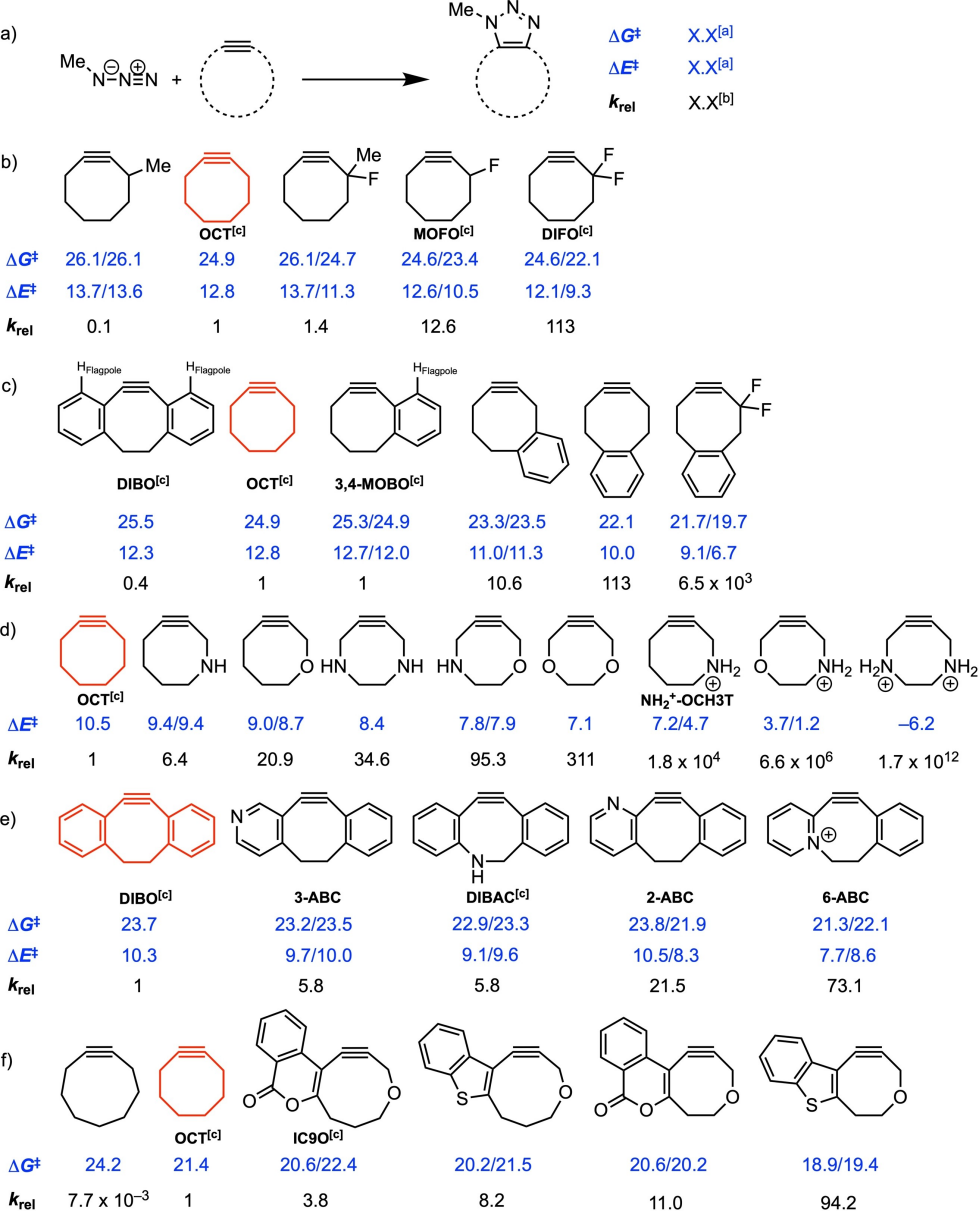
Gibbs free activation energies, electronic activation energies (blue; in kcal mol^−1^), and second‐order rate constants relative to cyclooctyne (black) for the a) 1,3‐DCA of methyl azide with b) exocyclic heterocyclooctynes (B3LYP/6‐311G(d,p);^[48]^ c) benzocyclooctynes (B3LYP/6‐311G(d,p);^[48]^ d) endocyclic heterocyclooctynes (B3LYP/6‐31G(d);^[49]^ e) dibenzocyclooctynes with different nitrogen placement (IEFPCM(water)‐M06‐2X/6‐311++G(d,p)), where *N*‐methylazidoacetamide is used as 1,3‐dipole and second‐order rate constants are relative to DIBO;^[50]^ f) benzoheterocyclooctynes (SMD(acetonitrile)‐B3LYP‐D3/6‐31++g(d,p)).^[51]^ [a] For 1,3‐DCA products that are regioisomeric cycloadducts, the Gibbs free activation energies and electronic activation energies are provided for the 1,4‐ and 1,5‐cycloadducts (1,4‐TS/1,5‐TS). [b] The relative second‐order rate constants (*k*_rel_) were calculated relative to the parent cyclooctyne, DIBO in Figure 7e, (*k*/*k*_parent_). If both the 1,4‐ and 1,5‐cycloadducts were formed, the *k*_rel_ was computed by using *k*_rel_=(*k*_1,4_+*k*_1,5_)/*k*_parent_. [c] This dipolarophile is both synthesized and utilized as a reagent in 1,3‐DCA reactions.

Goddard and coworkers investigated cyclooctyne‐based reagents bearing an exocyclic heteroatom (Figure 7b). They found that fluorination at the α‐position leads to a consistent enhancement in reactivity as the number of fluorine atoms increases from no F atoms (24.9 kcal mol^−1^) to one F atom (23.4 kcal mol^−1^) to two F atoms (22.1 kcal mol^−1^).^[48]^ This enhanced reactivity was again attributed to the hyperconjugative donation from the in‐plane alkyne π‐system into the σ*_C−F_ orbital, which directly leads to a stabilization of the transition state. On the other hand, this stabilizing hyperconjugative interaction was not present when a methyl group was placed on the α‐position of the cyclooctyne and hence an increased activation barrier (26.1 kcal mol^−1^) was observed. One can wonder whether this hyperconjugative interaction was the only reason for the enhanced reactivity or if other effects were also at play. The hyperconjugative donation should, in principle, be possible with other electron‐withdrawing groups, however, to which extent has not been investigated.

Another approach toward optimization of the SPAAC reaction would be to increase the predistortion of the dipolarophile (Figure 7c).^[48]^ It is expected that (bi)aryl substitution on the α‐ and β‐carbons would increase the predistortion and provide conjugation with the alkyne, resulting in enhanced SPAAC reactivity.^[38]^ However, the Gibbs free activation energy is for the biaryl substituted cyclooctyne higher than for the unsubstituted analog (unsubstituted: Δ*G* ^≠^=24.9 kcal mol^−1^, biaryl substituted: Δ*G* ^≠^=25.5 kcal mol^−1^). DFT calculations at B3LYP/6‐311G(d,p) revealed a steric repulsion between the incoming azide and the “flagpole” hydrogens on the aryl groups, which overrules the activation barrier‐lowering effect of the increased predistortion, ultimately, giving rise to a slightly higher activation barrier than for the unsubstituted cyclooctyne.^[48]^ Attachment of one aryl group will decrease both the steric repulsive and predistortive effects, resulting in a slightly enhanced reactivity (Δ*G* ^≠^=24.9 kcal mol^−1^) relative to biaryl‐substituted cyclooctyne. Nevertheless, the reactivity was equal to the unsubstituted cyclooctyne. As the aryl ring ‘walks’ over the ring, the activation barrier will decrease as the steric repulsion with the flagpole hydrogen also decreases (Δ*G* ^≠^=24.9 kcal mol^−1^→23.5 kcal mol^−1^→22.1 kcal mol^−1^). In addition, increased predistortion is present in the para‐substituted aryl ring, resulting in an enhanced reactivity. A combination of increased predistortion by the aryl group and electron‐withdrawing fluoride substituents on the α‐carbon will enhance the reactivity even more with an activation barrier of 19.7 kcal mol^−1^.

The effect of endocyclic heteroatoms on the 1,3‐DCA reactivity was investigated by Gold *et* *al*. (Figure 7d). They found that substitution of the propargylic carbon by a σ‐acceptor species enhanced the reactivity and proposed that the extent of the endocyclic activation is directly related to the acceptor capability of the activating group (NH_2_^+^ > O > NH).^[49]^ In the gas phase, this acceleration surpasses the effect of the exocyclic fluorine atoms. While the π_in‐plane_→σ*_C−F_ overlap in difluorinated cyclooctyne (DIFO) is comparable to the π_in‐plane_→σ*_C−N_ overlap in the cyclooctyne bearing one endocyclic NH_2_^+^ (NH_2_^+^‐OCT), the donation of π*_in‐plane_→σ*_C−F_ is much smaller than that of π*_in‐plane_→σ*_C−N_, due to a worse alignment (*gauche*) of the σ*_C−F_ orbital compared to the σ*_C−N_ orbital (*app*), allowing for greater assistance to the bond formation process in the latter situation. In the transition state, the C−N bond forming interactions were found to increase the population of the alkyne π*‐orbital, serving as a very efficient donor in the interaction with the stereoelectronically aligned (*app*) σ‐acceptors to stabilize the transition state and, ultimately, enhance the overall 1,3‐DCA reactivity.

Dones *et* *al*. explored the effects of nitrogen substitution in the DIBO scaffold on the 1,3‐DCA reactivity with *N*‐methylazidoacetamide (Figure 7e). They presented the first heterobiarylcyclooctynes (ABC) and found that these compounds showcased a synergy between the multiple electronic effects and increased predistortion.^[50]^ The reactivities of these ABCs were evaluated using DFT calculations at IEFPCM(water)‐M06‐2X/6‐311++G(d,p). For the 3‐, 4‐, 5‐ABCs, negligible differences in the reactivity were observed. Whereas, for 2‐ABC and 6‐ABC enhanced reactivity was observed, due to the propargylic C−N bond which is able to interact with the π‐system of the alkyne (π_in‐plane_→σ*_C−N_).^[46]^ Through activation strain analyses at the transition state structures, they found that the enhanced reactivity of 2‐ABC originates from a more stabilizing interaction energy and an increased predistortion. They found that 2‐ABC is able to benefit from additional intermolecular interactions with the 1,3‐dipole in the transition state, namely an interaction between the lone pair orbital on the aryl nitrogen and carbonyl π*‐acceptor orbital of the 1,3‐dipole (n_N_→π*_C=O_). Such an intermolecular interaction was, up till then, unprecedented in SPAAC reactions. Notably, 2‐ABC is the only ABC compound that favors the 1,5‐cycloadduct over the 1,4‐cycloadduct.

Balova and coworkers demonstrated an additional electronic effect that stabilizes the transition state as a consequence of out‐of‐plane bending of the fused heterocycle in the staggered conformation.^[51]^ They sought to develop novel SPAAC reagents that feature both heterocycle fusion and transition state stabilization via an endocyclic σ‐acceptors with an *app* arrangement. They fused eight‐ and nine‐membered heteroalkynes to both benzothiophene and isocoumarin (Figure 7f). The reactivities of these novel 1,3‐DCA reagents were investigated using DFT calculations at SMD(MeCN)‐B3LYP‐D3/6‐31++g(d,p). The fused heterocycles exhibited a small HOMO–LUMO gap of approximately 5 eV. Notably, on going from eight‐ to nine‐membered rings, the reactivities of these species were only reduced by less than one order of magnitude, whereas this is three orders of magnitude for non‐substituted cycloalkynes.^[52]^ Activation strain analyses at the transition state suggested that the increased activation strain in the less predistorted nine‐membered fused heterocycles was offset in the staggered conformation, but, in contrast not in the eclipsed conformation. The more stabilizing interaction energies of the nine‐membered fused heterocycloalkynes in the staggered conformation were attributed to both the interaction between the in‐plane π‐system and σ*_C−X_ and the interaction between the in‐plane π‐system and the out‐of‐plane π*_Het_ of the heterocycle. As the orbital alignment is improved in the out‐of‐plane bent conformer, the overlap for π*_Het_→π*_in‐plane_ interaction is enhanced. Thus, the preference for the staggered conformer of the nine‐membered fused heterocycles allows for transition state stabilization through π*_in‐plane_→σ*_C−X_ and π*_Het_→π*_in‐plane_ interactions, which causes the loss in reactivity compared to the eight‐membered fused heterocycle to be only little.

## 5 Bioorthogonal 1,3‐Dipolar Cycloadditions

**Scheme 9:**
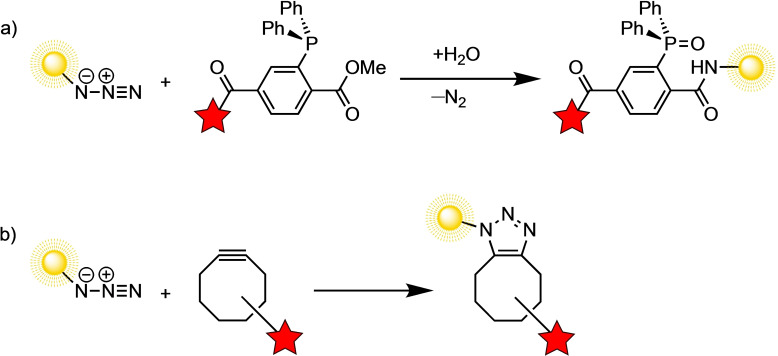
The schematic representation of bioorthogonal labeling of biomolecules. The azide is a chemical reporter to which the cyclooctyne can selectively ligate through the a) Staudinger reaction or b) strain‐promoted azide‐alkyne cycloaddition (SPAAC).

In 1997, the seminal work of Bertozzi and coworkers showed how unnatural monosaccharides on the cell surface could act as chemical reporter groups for covalent ligation with a hydrazide functional group under physiological conditions without perturbing the native environment.^[53]^ A few years later, the same group demonstrated that azides on the cell surface could also function as chemical reporter groups for the Staudinger reaction (Scheme 9a).^[54]^ As both azides and phosphines are abiotic in and outside the cell, the Staudinger ligation allowed for intracellular application. This bioorthogonal strategy, facilitated by selective chemical reactions within the cellular environment, allowed for probing previously unobservable intracellular activity and showed potential for noninvasive imaging and therapeutic targeting (Scheme 9).

Bioorthogonal reactions are extremely fast and can proceed in living systems without perturbing native biochemical processes. These reactions should be selective and orthogonal to functional groups in the biological environment, should not produce any (harmful) byproducts, and must not depend on auxiliary molecules. 1,3‐DCA reactions are rare in biochemistry, since azides and alkynes did not show reactivity towards biological molecules in the reaction condition within the cell, and no enzymes have been identified to date that catalyze this class of reaction *in vivo*.[^36^, ^55^] Azides and alkynes are, however, readily introduced into organic compounds and are, therefore, ideal functional groups to be included on the target biomolecules through a metabolic pathway to construct chemical reporters for chemical bioconjugation. The CuAAC reaction, for example, was found to be a useful tool in many complex *in vitro* labeling problems.^[56]^

Due to the presence of copper and its toxicity to both bacterial and mammalian cells, CuAAC methods were impractical for most *in vivo* applications.[^36^, ^56b^] As an alternative, Bertozzi and coworkers utilized the SPAAC reaction to circumvent the problem of copper toxicity (Scheme 9b).^[36]^ They showcased the SPAAC reaction for the selective modification of biomolecules *in vitro* and in living cells without apparent physical harm or toxicity. This led to the successful incorporation of azido‐substituted galactose amine derivatives into living zebrafish embryos.^[57]^ These glycans were desirable targets as they are markers for altered gene expression during development and disease progression. The cyclooctyne substituted fluorescent probes were selectively ligated to the azide reporter molecules, which enabled *in vivo* imaging at subcellular resolution during development. This allowed for high detail spatiotemporal imaging during the development of the zebrafish, which would have been unattainable with alternative, more conventional methods.

**Figure 8:**
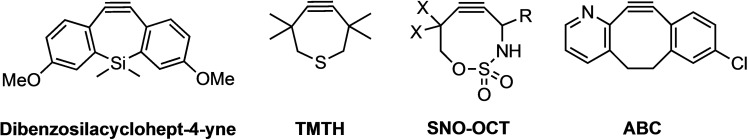
Cycloalkyne reagents that have been rationally designed and synthesized to increase the reactivity towards the 1,3‐DCA with azide derivatives.

*In vivo* applications of bioorthogonal reactions require high chemoselectivity and second‐order rate constants exceeding 1 M^−1^ s^−1^ because of the micromolar concentrations in the cellular environment. The design principles presented in Section 4 of this review article have been excessively used to design novel SPAAC reagents. The Bertozzi group introduced a stable seven‐membered cycloalkyne for bioorthogonal application, 3,3,6,6‐tetramethylthiacycloheptane (TMTH, Figure 8).^[58]^ They reported second‐order rate constants of 4.0 M^−1^ s^−1^ for the 1,3‐DCA reaction with benzyl azide in acetonitrile. They suggested that introducing an endocyclic heteroatom would decrease the predistortion of the seven‐membered cycloalkyne and resulted in a non‐transient reagent. The applicability of TMTH was demonstrated through the successful selective labeling of azide functionalized proteins, although the reaction yield was lower than expected. Martínek *et* *al*. demonstrated the highest rate constant reported for the SPAAC reaction for the 1,3‐DCA between benzyl azide and dibenzosilacyclohept‐4‐yne (Figure 8) in both methanol and acetonitrile, namely, 20 M^−1^ s^−1^.^[59]^ The highly predistorted seven‐membered cycloalkyne showed remarkable stability, also in an aqueous environment. Nevertheless, to date, seven‐membered cycloalkyne reagents have not been applied for 1,3‐DCA reactions *in vitro* or *in vivo*.

Schomaker and coworkers designed sulfur‐, nitrogen‐, and oxygen‐containing heterocyclic cyclooctyne (SNO‐OCT, Figure 8) derivatives that are versatile reagents for bioorthogonal application.^[60]^ SNO‐OCT derivatives exploit electronic effects through both endo and exocyclic heteroatoms. Specifically, the endocyclic nitrogen is suggested to function as a σ*_C−N_ acceptor in the *app* arrangement, which allows for hyperconjugative assistance. The sulfonyl group increases the acceptor ability of the propargylic heteroatom emphasizing the electronic effects of the σ*_C−N_ acceptor. The second‐order rate constant for the 1,3‐DCA reaction with benzyl azide in acetonitrile was 0.026 M^−1^ s^−1^ and could be increased to 0.13 M^−1^ s^−1^ in polar solvents, such as water. The SNO‐OCT was successfully applied in a bioorthogonal context, through efficient bioconjugation to azide‐PEG3‐biotin.

In recent years, the thermal 1,3‐DCA reaction had been used in the context of sulfur(VI) fluoride exchange (SuFEx) click chemistry, which could be relevant for sulfonyl linkage in biologically active compounds and late‐stage diversification.^[61]^ 1,3‐dipoles had shown to undergo the 1,3‐DCA reaction with ethenylsulfonyl (ESF) or 1‐bromoethene‐1‐sulfonyl fluoride (Br‐ESF) yielding sulfonyl fluoride‐substituted pyrazoles and pyrazolines.^[62]^ Furthermore, diazoacetamide reacts at ambient temperatures while azides do not, thus broadening the scope and diversity of potential substrates for this reaction.^[63]^ Relative to the azido, diazo compounds feature a higher HOMO_1,3‐dipole_ that could engage in more favorable NED HOMO_1,3‐dipole_–LUMO_dipolarophile_ interaction. The authors substantiated this through an activation strain analysis (distortion/interaction analysis)[^12^, ^15^] of the transition state structures, which suggested that the enhanced reactivity arises from an increased stabilizing interaction in the transition state. NBO analysis revealed that the NED HOMO_1,3‐dipole_–LUMO_dipolarophile_ interaction was, indeed, the dominant interaction.^[64]^ In addition, it was shown that the transition state was stabilized through π*_in‐plane_→σ*_S−F_ interactions, which provides delocalization of the partially filled π*_dipolarophile_ orbital, because of the directional charge transfer. The authors introduced a chemoselective 1,3‐DCA reaction between ESF‐containing dipolarophiles and 1,3‐dipoles to synthesize useful building blocks for modular SuFEx click chemistry.

The next challenge was the design of mutual orthogonal bioorthogonal reaction pairs to facilitate the simultaneous imagining of multiple biomolecules. By exploiting the steric demand of distinct cyclooctyne derivatives Svatunek *et* *al*. demonstrated access to chemoselective labeling using only the SPAAC reaction.^[65]^ The reactivities of primary, secondary, and tertiary azides towards two cyclooctynes, BCN, and the more sterically congested ADIBO were investigated. While the reactivities of primary, secondary, and tertiary azides with BCN as well as the primary and secondary azides with ADIBO are similar, the second‐order rate constant is five orders of magnitude smaller for the reaction between the tertiary azide and ADIBO. By employing activation strain and energy decomposition analyses,[^12^, ^15^] the authors found that the increase in reaction barrier was caused by less favorable interaction energy, which, in turn, was traced back to the more destabilizing Pauli repulsion between the *tert*‐butyl group on the tertiary azide and ADIBO. The chemoselectivity was exploited through the highly selective semiorthogonal dual‐labeling with the exclusive use of the SPAAC reaction.

Additionally, the reactivity of different 1,3‐dipoles can be exploited in the design of mutual orthogonal bioorthogonal reaction pairs. Diazo compounds have been shown to chemoselectively react in the presence of azido compound in a bioorthogonal manner.[^63^, ^66^] Aranoff *et* *al*. demonstrated chemoselective 1,3‐DCA between a diazo compound and a terminal alkene in the presence of azido groups.^[66c]^ Because of the high nucleophilicity of diazo 1,3‐dipole, the enhanced NED HOMO_1,3‐dipole_–LUMO_dipolarophile_ interaction alleviates the necessity of predistortion of the dipolarophile for obtaining 1,3‐DCA rates desired for bioorthogonal chemistry.[^66c^, ^66e^]

Dones *et* *al*. presented the heterobiarylcyclooctynes (ABC, Figure 8) that feature fused cyclooctyne rings and pyridine with the nitrogen on the 2‐position.^[50]^ They observed a 1200 times enhancement of the second‐order rate constant for the 1,3‐DCA addition between *N*‐methyldiazoacetamide and ABC relative to DIBO, whereas only a 30 times acceleration for *N*‐methylazidoacetamide. On top of LUMO lowering effect, they found the enhanced reactivity could be sourced to a stronger hydrogen bonding interaction between ABC and the *N*‐methyldiazoacetamide (N⋅⋅⋅H−N) relative to the significantly weaker intermolecular interaction with *N*‐methylazidoacetamide (N⋅⋅⋅C=O). Both these additional interactions are not present when DIBO is used as dipolarophile (*vide supra*), resulting in the observed suppressed 1,3‐DCA reactivity. These factors can be exploited in mutual orthogonal bioorthogonal chemistry.

Coelho and coworkers presented a multivariate regression model to predict the second‐order rate constants for 1,3‐DCAs and inverse electron demand Diels‐Alder (IEDDA) reactions and employed these in identifying mutually orthogonal bioorthogonal reaction pairs.^[67]^ They parametrized 1,3‐DCA reactions using molecular descriptors after reagents optimization at M06‐2X/6‐31G(d). The descriptors included features such as distances, bond angles, dihedral angles, vibration frequencies and intensities, NBO charges, HOMO/LUMO energies, and the Sterimol parameters, which were developed to quantify the steric demand along individual principal axes.^[68]^ Using these parameters in multivariate regression models, the authors constructed five datasets with *R* ^2^ values in the range of 0.74 to 0.94. The same method was applied to the IEEDA reactants.^[67b]^ With the two datasets in hand, they constructed a matrix of the predicted Δ*G* ^≠^ of the respective reactants. The selected reaction pairs were identified based on differences in Δ*G* ^≠^. The group reported the successful prediction of four mutually orthogonal bioorthogonal reaction pairs, of which one was previously reported.^[69]^ This nicely showed how statistical modeling can play a key role in the discovery of mutually orthogonal bioorthogonal reaction pairs.

More recently, the groups of Raines and Schomaker combined computational and experimental efforts to design triple mutually orthogonal bioorthogonal reaction pairs.^[70]^ Through design principles that were established in earlier studies and assisted by computational methods, they designed a novel heterocyclic cycloalkyne that would display orthogonal reactivities.[^60^, ^63^] Maegawa *et* *al*. employed three distinct bioorthogonal reactions, namely the Staudinger ligation, SPAAC, and CuAAC, to showcase the triple mutual orthogonal site‐selective ligation onto azide functional groups.^[71]^ As more reactions are being added to the bioorthogonal library and the design principles become better formulated, more elaborate bioorthogonal strategies can be formulated, which will open up new opportunities for *in vivo* experimentation.^[72]^

## 6 Conclusion

1,3‐Dipolar cycloadditions (1,3‐DCA) have emerged as one of the quintessential reactions in organic, material, and biological chemistry. In 1971, Sustmann described, based on the work of Fukui, the reactivity of a phenyl azide and a set of dipolarophiles by means of frontier molecular orbital (FMO) interactions. Since then, significant progress has been made in improving the understanding of the physical factors that determine the reactivity and selectivity of 1,3‐DCAs. In this review, we have highlighted all of the major advancements towards rationalizing the various factors influencing the reactivity of 1,3‐DCAs, starting from the original conception of the reaction, all the way to state‐of‐the‐art quantum chemical calculations and modern experimental efforts. We expect our insights to be useful as a guide to the design and synthesis of novel 1,3‐DCA reaction pairs, which can be applied in a wide‐range of chemistries.

The reactivity of the thermal 1,3‐DCA reaction can be predicted and explained by the normal electron demand (NED), HOMO_1,3‐dipole_–LUMO_dipolarophile_, and the inverse electron demand (IED), LUMO_1,3‐dipole_–HOMO_dipolarophile_, orbital interactions. This thermal reaction does, however, require elevated temperatures making it, in general, sluggish, and unselective. The reactivity of 1,3‐DCAs can be enhanced, either through catalysis or strain‐promoted reactivity. Efficient regioselective conversion to the 1,4‐ and the 1,5‐cycloadduct has been achieved by copper‐catalyzed azide‐alkyne cycloaddition (CuAAC) or ruthenium‐catalyzed azide‐alkyne cycloaddition (RuAAC), respectively. Various other transition metal catalysts (*i. a*. nickel, zinc, iron) have also been shown to effectively accelerate the 1,3‐DCA reaction.

A metal‐free alternative to enhance the 1,3‐DCA reactivity is the strain‐promoted azide‐alkyne cycloaddition (SPAAC) reaction, where the dipolarophile is predistorted towards the transition state geometry. This predistortion of the dipolarophile not only reduces the activation strain, but also enhances the stabilizing orbital interactions, since predistortion lowers the HOMO–LUMO gap of the dipolarophile, thereby, reducing the NED and IED orbital energy gaps with the dipole. Additionally, predistortion gives rise to a larger orbital amplitude at the external face of the π‐system amplifying the NED and IED orbital overlaps. The rate of SPAAC reaction can be further accelerated by stabilization of the transition state, which can be accomplished via three distinct mechanisms: i) hyperconjugative assistance to bending (π_in‐plane_→σ*_C−X_); ii) hyperconjugative assistance to bond formation (π _in‐plane_→σ*_C−X_ and π*_in‐plane_→σ*_C−X_); and iii) the electrostatic interactions between the exocyclic heteroatom of the dipolarophile and a substituent on the 1,3‐dipole. The SPAAC reaction has especially gained attention in the context of bioorthogonal chemistry, where it allows for the labeling of biomolecules under physiological conditions.

Throughout the years, critical physical factors that determine the reactivity of 1,3‐DCAs have been identified through quantum chemical calculations. The understanding of these physical factors offers opportunities for even more elaborate design principles, both in optimizing 1,3‐DCA reaction pairs and in finding mutually orthogonal bioorthogonal reaction pairs. The upcoming field of data science will have a pivotal role to play in finding more advanced mutually orthogonal techniques, thereby, expanding the bioorthogonal toolbox.

## Conflict of interest

There are no conflicts to declare.

## Biographical Information

*Steven E. Beutick (Amsterdam, NL, 1996) obtained his B.Sc. and M.Sc. in the joined degree program of the University of Amsterdam (UvA) and the Vrije Universiteit (VU) Amsterdam and carried out his M.Sc. research under the supervision of Dr. Trevor A. Hamlin and Prof. Dr. Evert Jan Meijer. In 2021, he joined the University of Padova in collaboration with the VU Amsterdam for a Ph.D. project under the supervision of Prof. Dr. Laura Orian and Prof. Dr. F. Matthias Bickelhaupt. The topic of his Ph.D. research is on the reactivity and selectivity of reactions for protein functionalization and bioorthogonal chemistry*.

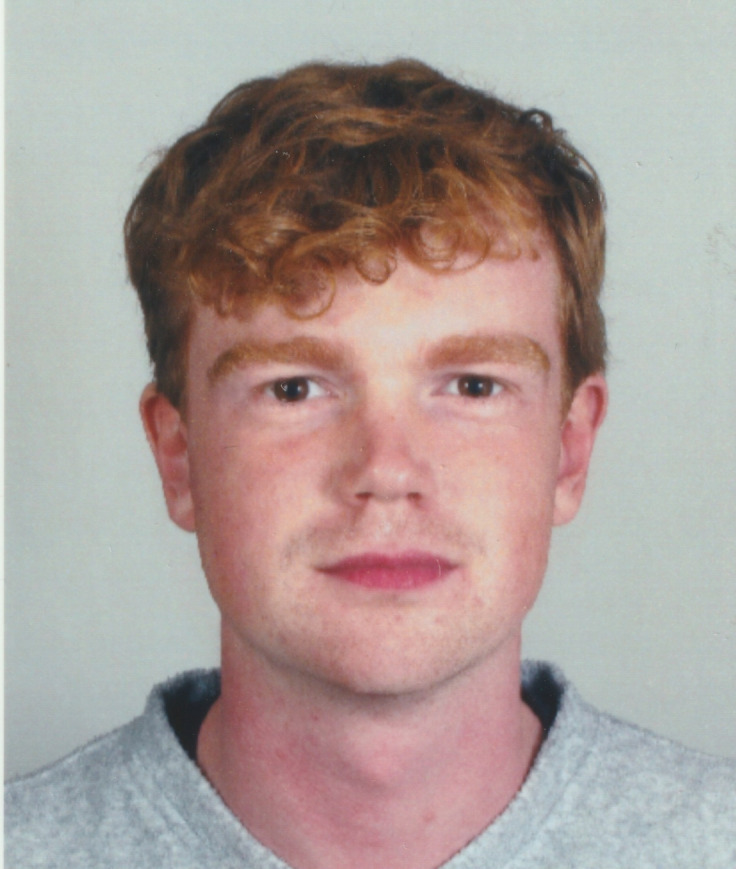

## Biographical Information

*Pascal Vermeeren (Amsterdam, NL, 1993) received his M.Sc. (2017) in Chemistry from the Vrije Universiteit (VU) Amsterdam. In 2022, he completed his Ph.D. (cum laude) under the supervision of Prof. Dr. F. Matthias Bickelhaupt and Dr. Trevor A. Hamlin in the Department of Theoretical Chemistry at VU Amsterdam. At present, he is a postdoctoral researcher in the groups of Prof. Dr. F. Matthias Bickelhaupt and Dr. Trevor A. Hamlin. His scientific interests include elucidating the role of steric Pauli repulsion in chemical reactivity and molecular structures*.

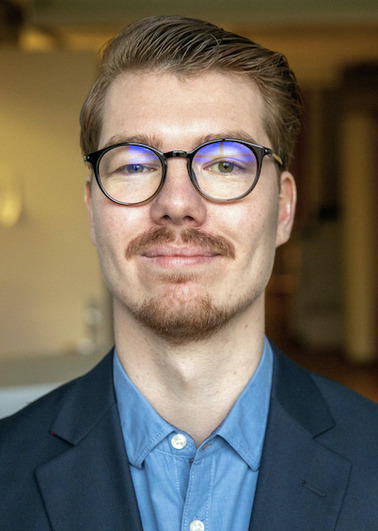

## Biographical Information

*Trevor A. Hamlin (Fountain Valley, California, USA, 1988) received his B.S. (2010) in biochemistry from Albright College and Ph.D. (2015) in chemistry from the University of Connecticut. Following this, he carried out his postdoctoral training (2015–2019) in the group of Prof. Dr. F. Matthias Bickelhaupt at the Vrije Universiteit (VU) Amsterdam. In 2019, he joined the Theoretical Chemistry department at VU Amsterdam where he is currently an Assistant Professor (with tenure). His primary research interests are centered on unravelling reaction mechanisms and developing unified concepts to control the reactivity and selectivity of chemical transformations*.

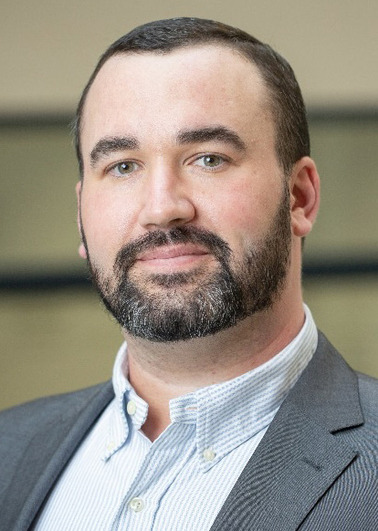

## References

[1a] R. Huisgen , Proc. Chem. Soc. London 1961, 357–369;

[1b] R. Huisgen , Angew. Chem. Int. Ed. Engl. 1963, 2, 565–598;

[1c] R. Huisgen , Angew. Chem. Int. Ed. Engl. 1963, 2, 633–645;

[2] V. V. Rostovtsev , L. G. Green , V. V. Fokin , K. B. Sharpless , Angew. Chem. Int. Ed. 2002, 41, 2596–2599; 10.1002/1521-3773(20020715)41:14<2596::AID-ANIE2596>3.0.CO;2-4 12203546

[3a] J. P. Collman , N. K. Devaraj , C. E. D. Chidsey , Langmuir 2004, 20, 1051–1053; 15803676 10.1021/la0362977 PMC3428800

[3b] A. E. Speers , G. C. Adam , B. F. Cravatt , J. Am. Chem. Soc. 2003, 125, 4686–4687; 12696868 10.1021/ja034490h

[3c] P. L. Golas , N. V. Tsarevsky , B. S. Sumerlin , K. Matyjas-Zewski , Macromolecules 2006, 39, 6451–6457;

[3d] A. Krasiński , Z. Radić , R. Manetsch , J. Raushel , P. Taylor , K. B. Sharpless , H. C. Kolb , J. Am. Chem. Soc. 2005, 127, 6686–6692; 15869290 10.1021/ja043031t

[3e] G. C. Tron , T. Pirali , R. A. Billington , P. L. Canonico , G. Sorba , A. A. Genazzani , Med. Res. Rev. 2008, 28, 278–308. 17763363 10.1002/med.20107

[4] E. Buchner , Ber. Dtsch. Chem. Ges. 1888, 21, 2637–2647.

[5] K. von Auwers , F. König , Justus Liebigs Ann. Chem. 1932, 496, 27–51.

[6a] K. Fukui , T. Yonezawa , H. Shingu , J. Chem. Phys. 1952, 20, 722–725;

[6b] K. Fukui , T. Yonezawa , C. Nagata , H. Shingu , J. Chem. Phys. 1954, 22, 1433–1442.

[7a] R. Sustmann , Tetrahedron Lett. 1971, 12, 2717–2720;

[7b] R. Sustmann , H. Trill , Angew. Chem. Int. Ed. Engl. 1972, 11, 838–840;

[7c] R. Sustmann , Pure Appl. Chem. 1974, 40, 569–593;

[7d] J. Geittner , R. Huisgen , R. Sustmann , Tetrahedron Lett. 1977, 18, 881–884.

[8] T. Koopmans , Physica 1934, 1, 104–113.

[9] P.-P. Chen , P. Ma , X. He , D. Svatunek , F. Liu , K. N. Houk , J. Org. Chem. 2021, 86, 5792–5804. 33769821 10.1021/acs.joc.1c00239 PMC8154615

[10] F. S. Vilhena , F. M. Bickelhaupt , J. W. M. Carneiro , Eur. J. Org. Chem. 2017, 4313–4318.

[11a] D. H. Ess , K. N. Houk , J. Am. Chem. Soc. 2007, 129, 10646–10647; 17685614 10.1021/ja0734086

[11b] D. H. Ess , K. N. Houk , J. Am. Chem. Soc. 2008, 130, 10187–10198. 18613669 10.1021/ja800009z

[12a] F. M. Bickelhaupt , K. N. Houk , Angew. Chem. 2017, 129, 10204; 10.1002/anie.201701486 PMC5601271 28447369

[12b] P. Vermeeren , T. A. Hamlin , F. M. Bickelhaupt , Chem. Commun. 2021, 57, 5880–5896; 10.1039/d1cc02042k PMC8204247 34075969

[12c] P. Vermeeren , S. C. C. van der Lubbe , C. Fonseca Guerra , F. M. Bickelhaupt , T. A. Hamlin , Nat. Protoc. 2020, 15, 649–667. 31925400 10.1038/s41596-019-0265-0

[13] T. A. Hamlin , D. Svatunek , S. Yu , L. Ridder , I. Infante , L. Visscher , F. M. Bickelhaupt , Eur. J. Org. Chem. 2019, 378–386.

[14] F. Zapata , L. Ridder , J. Hidding , C. R. Jacob , I. Infante , L. Visscher , J. Chem. Inf. Model. 2019, 59, 3191–3197. 31260292 10.1021/acs.jcim.9b00384 PMC6651270

[15] For an overview of the EDA method, see:

[15a] T. A. Hamlin, P. Vermeeren, C. Fonseca Guerra, F. M. Bickelhaupt, in *Complementary Bonding Analysis*, (Ed. S. Grabowsky), De Gruyter, Berlin, **2021**, pp. 199–212; for a detailed overview of the EDA method, see:

[15b] F. M. Bickelhaupt, E. J. Baerends, in *Reviews in Computational Chemistry*, (Eds. K. B. Lipkowitz, D. B. Boyd), Wiley, Hoboken, **2000**, pp. 1–86.

[16a] K. N. Houk , Acc. Chem. Res. 1975, 8, 361–369;

[16b] I. Fleming , J. P. Michael , L. E. Overman , G. F. Taylor , Tetrahedron Lett. 1978, 19, 1313–1316.

[17] S. Yu , P. Vermeeren , K. van Dommelen , M. Bickelhaupt , T. A. Hamlin , Chem. Eur. J. 2020, 26, 11529–11539. 32220086 10.1002/chem.202000857 PMC7540365

[18] Y.-R. Luo, *Comprehensive Handbook of Chemical Bond Energies*, CRC Press, Boca Raton, FL, USA **2007**.

[19] H. C. Kolb , M. G. Finn , K. B. Sharpless , Angew. Chem. Int. Ed. 2001, 40, 2004–2021; 10.1002/1521-3773(20010601)40:11<2004::AID-ANIE2004>3.0.CO;2-5 11433435

[20] C. W. Tornøe , C. Christensen , M. Meldal , J. Org. Chem. 2002, 67, 3057–3064. 11975567 10.1021/jo011148j

[21] J. C. Jewett , C. R. Bertozzi , Chem. Soc. Rev. 2010, 39, 1272–1279. 20349533 10.1039/b901970g PMC2865253

[22] J. E. Hein , V. V. Fokin , Chem. Soc. Rev. 2010, 39, 1302–1315. 20309487 10.1039/b904091a PMC3073167

[23] L. Zhu , C. J. Brassard , X. Zhang , P. M. Guha , R. J. Clark , Chem. Rec. 2016, 1501–1517. 27216993 10.1002/tcr.201600002

[24a] C. Nolte , P. Mayer , B. F. Straub , Angew. Chem. Int. Ed. 2007, 46, 2101–2103; 10.1002/anie.200604444 17300119

[24b] V. O. Rodionov , S. I. Presolski , S. Gardinier , Y. H. Lim , M. G. Finn , J. Am. Chem. Soc. 2007, 129, 12696–12704. 17914816 10.1021/ja072678l

[25] B. T. Worrell , J. A. Malik , V. V. Fokin , Science 2013, 340, 457–460. 23558174 10.1126/science.1229506 PMC3651910

[26a] M. Ahlquist , V. V. Fokin , Organometallics. 2007, 26, 4389–4391;

[26b] V. O. Rodionov , V. V. Fokin , M. G. Finn , Angew. Chem. Int. Ed. 2005, 44, 2210–2215; 10.1002/anie.200461496 15693051

[26c] V. O. Rodionov , S. I. Presolski , D. D. Díaz , V. V. Fokin , M. G. Finn , J. Am. Chem. Soc. 2007, 129, 12705–12712; 17914817 10.1021/ja072679d

[26d] S. I. Presolski , V. Hong , S.-H. Cho , M. G. Finn , J. Am. Chem. Soc. 2010, 132, 14570–14576. 20863116 10.1021/ja105743g PMC2956586

[27] B. F. Straub , Chem. Commun. 2007, 3868–3870. 10.1039/b706926j 18219789

[28] J. Héron , D. Balcells , ACS Catal. 2022, 12, 4744–4753.

[29a] L. Zhang , X. Chen , P. Xue , H. H. Y. Sun , I. D. Williams , K. B. Sharpless , V. V. Fokin , G. Jia , J. Am. Chem. Soc. 2005, 127, 15998–15999; 16287266 10.1021/ja054114s

[29b] L. K. Rasmussen , B. C. Boren , V. V. Fokin , Org. Lett. 2007, 9, 5337–5339; 18052070 10.1021/ol701912s

[29c] B. C. Boren , S. Narayan , L. K. Rasmussen , L. Zhang , H. Zhao , Z. Lin , G. Jia , V. V. Fokin , J. Am. Chem. Soc. 2008, 130, 8923–8930. 18570425 10.1021/ja0749993

[30] M. Lamberti , G. C. Fortman , A. Poater , J. Broggi , A. M. Z. Slawin , L. Cavallo , S. P. Nolan , Organometallics 2012, 31, 756–767.

[31] C. D. Smith , M. F. Greaney , Org. Lett. 2013, 15, 4826–4829. 24001177 10.1021/ol402225d PMC4331842

[32] L. Hong , W. Lin , F. Zhang , R. Liu , X. Zhou , Chem. Commun. 2013, 49, 5589–5591. 10.1039/c3cc42534g 23676902

[33] W. G. Kim , M. E. Kang , J. B. Lee , M. H. Jeon , S. Lee , J. Lee , B. Choi , P. M. S. D. Cal , S. Kang , J. Kee , G. J. L. Bernardes , J. Rohde , W. Choe , S. Y. Hong , J. Am. Chem. Soc. 2017, 139, 12121–12124. 28814075 10.1021/jacs.7b06338

[34a] K. V. Gothelf , K. Anker Jørgensen , Chem. Commun. 2000, 1449–1458;

[34b] L. M. Stanley M P Sibi , Chem. Rev. 2008, 108, 2887–2902; 18613728 10.1021/cr078371m

[34c] R. Narayan , M. Potowski , Z.-J. Jia , A. P. Antonchick , H. Waldmann , Acc. Chem. Res. 2014, 47, 1296–1310; 24730692 10.1021/ar400286b PMC4004623

[34d] C. Nájera , M. de Gracia Retamosa , J. M. Sansano , A. de Cózar , F. P. Cossío , Eur. J. Org. Chem. 2007, 5038–5049;

[34e] A. Pascual-Escudero , A. de Cózar , F. P. Cossío , J. Adrio , J. C. Carretero , Angew. Chem. Int. Ed. 2016, 55, 15334–15338; 10.1002/anie.201609187 27862800

[34f] G. S. Caleffi , O. Larrañaga , M. Ferrándiz-Saperas , P. R. R. Costa , C. Nájera , A. de Cózar , F. P. Cossío , J. M. Sansano , J. Org. Chem. 2019, 84, 10593–10605. 31348647 10.1021/acs.joc.9b00267

[35a] G. Wittig , A. Krebs , Chem. Ber. 1961, 94, 3260–3275;

[35b] A. T. Blomquist , L. H. Liu , J. Am. Chem. Soc. 1953, 75, 2153–2154.

[36] N. J. Agard , J. A. Prescher , C. R. Bertozzi , J. Am. Chem. Soc. 2004, 126, 15046–15047. 15547999 10.1021/ja044996f

[37] J. M. Baskin , J. A. Prescher , S. T. Laughlin , N. J. Agard , P. V. Chang , I. A. Miller , A. Lo , J. A. Codelli , C. R. Bertozzi , Proc. Natl. Acad. Sci. USA 2007, 104, 16793–17797. 17942682 10.1073/pnas.0707090104 PMC2040404

[38] X. Ning , J. Guo , M. A. Wolfert , G.-J. Boons , Angew. Chem. Int. Ed. 2008, 47, 2253–2255; 10.1002/anie.200705456 PMC2835304 18275058

[39] H. Meier , C. Schuh-Popitz , H. Peiersen , Angew. Chem. Int. Ed. Engl. 1981, 20, 270–271.

[40] J. Dommerholt , S. Schmidt , R. Temming , L. J. A. Hendriks , F. P. J. T. Rutjes , J. C. M. van Hest , D. J. Lefeber , P. Friedl , F. L. van Delft , Angew. Chem. Int. Ed. 2010, 49, 9422–9425; 10.1002/anie.201003761 PMC3021724 20857472

[41] J. C. Jewett , E. M. Sletten , C. R. Bertozzi , J. Am. Chem. Soc. 2010, 132, 3688–3690. 20187640 10.1021/ja100014q PMC2840677

[42] J. Dommerholt , O. van Rooijen , A. Borrmann , C. Fonseca Guerra , F. M. Bickelhaupt , F. L. van Delft , Nat. Commun. 2014, 5, 5378. 25382411 10.1038/ncomms6378

[43a] S. Xie , S. A. Lopez , O. Ramström , M. Yan , K. N. Houk , J. Am. Chem. Soc. 2015, 137, 2958–2966; 25553488 10.1021/ja511457g PMC4351169

[43b] S. Xie , M. Sundhoro , K. N. Houk , M. Yan , Acc. Chem. Res. 2020, 53, 937–948. 32207916 10.1021/acs.accounts.0c00046

[44] T. A. Hamlin , B. J. Levandowski , A. K. Narsaria , K. N. Houk , F. M. Bickelhaupt , Chem. Eur. J. 2019, 25, 6342–6348. 30779472 10.1002/chem.201900295 PMC6519225

[45a] R. W. Strozier , P. Caramella , K. N. Houk , J. Am. Chem. Soc. 1979, 101, 1340–1343;

[45b] N. G. Rondan , L. N. Domelsmith , K. N. Houk , A. T. Bowne , R. H. Levin , Tetrahedron Lett. 1979, 20, 3237–3240;

[45c] D. M. Hoffman , R. Hoffmann , C. R. Fisel , J. Am. Chem. Soc. 1982, 104, 3858–3875.

[46] B. Gold , N. E. Shevchenko , N. Bonus , G. B. Dudley , I. V. Alabugin , J. Org. Chem. 2012, 77, 75–89. 22077877 10.1021/jo201434w

[47] I. V. Alabugin , T. A. Zeidan , J. Am. Chem. Soc. 2002, 124, 3175–3185. 11902907 10.1021/ja012633z

[48] K. Chenoweth , D. Chenoweth , W. A. Goddard , Org. Biomol. Chem. 2009, 7, 5255–5258. 20024122 10.1039/b911482c

[49] B. Gold , G. B. Dudley , I. V. Alabugin , J. Am. Chem. Soc. 2013, 135, 1558–1569. 23272641 10.1021/ja3114196

[50] J. M. Dones , N. S. Abularrage , N. Khanal , B. Gold , R. T. Raines , J. Am. Chem. Soc. 2021, 143, 9489–9497. 34151576 10.1021/jacs.1c03133 PMC11753763

[51] N. A. Danilkina , A. I. Govdi , A. F. Khlebnikov , A. O. Tikhomirov , V. V. Sharoyko , A. A. Shtyrov , M. N. Ryazantsev , S. Bräse , I. Balova , J. Am. Chem. Soc. 2021, 143, 16519–16537. 34582682 10.1021/jacs.1c06041

[52] E. M. Sletten , G. de Almeida , C. R. Bertozzi , Org. Lett. 2014, 16, 1634–1637. 24588780 10.1021/ol500260d PMC3993865

[53] L. K. Mahal , K. J. Yarema , C. R. Bertozzi , Science 1997, 276, 1125–1128. 9173543 10.1126/science.276.5315.1125

[54] E. Saxon , C. R. Bertozzi , Science 2000, 287, 2007–2010. 10720325 10.1126/science.287.5460.2007

[55] E. H. Krenske , A. Patel , K. N. Houk , J. Am. Chem. Soc. 2013, 135, 17638–17642. 24195703 10.1021/ja409928z

[56a] A. E. Speers , G. C. Adam , B. F. Cravatt , J. Am. Chem. Soc. 2003, 125, 4686–4687; 12696868 10.1021/ja034490h

[56b] T. S. Seo , Z. Li , H. Ruparel , J. Ju , J. Org. Chem. 2003, 68, 609–612; 12530893 10.1021/jo026615r

[56c] A. J. Link , D. A. Tirrell , J. Am. Chem. Soc. 2003, 125, 11164–11165; 16220915 10.1021/ja036765z

[56d] Q. Wang , T. R. Chan , R. Hilgraf , V. V. Fokin , K. B. Sharpless , M. G. Finn , J. Am. Chem. Soc. 2003, 125, 3192–3193. 12630856 10.1021/ja021381e

[57] S. T. Laughlin , J. M. Baskin , S. L. Amacher , C. R. Bertozzi , Science 2008, 320, 664–667. 18451302 10.1126/science.1155106 PMC2701225

[58] G. de Almeida , E. M. Sletten , H. Nakamura , K. K. Palaniappan , C. R. Bertozzi , Angew. Chem. Int. Ed. 2012, 51, 2443–2447; 10.1002/anie.201106325 PMC3384729 22282228

[59] M. Martínek , L. Filipová , J. Galeta , L. Ludvíková , P. Klán , Org. Lett. 2016, 18, 4892–4895. 27624804 10.1021/acs.orglett.6b02367

[60a] E. G. Burke , B. Gold , T. T. Hoang , R. T. Raines , J. M. Schomaker , J. Am. Chem. Soc. 2017, 139, 8029–8037; 28505435 10.1021/jacs.7b03943 PMC5548293

[60b] E. G. Burke , J. M. Schomaker , J. Org. Chem. 2017, 82, 9038–9046. 28795808 10.1021/acs.joc.7b01506

[61a] J. Dong , L. Krasnova , M. G. Finn , K. B. Sharpless , Angew. Chem. Int. Ed. 2014, 53, 9430–9448; 10.1002/anie.201309399 25112519

[61b] Z. Liu , J. Li , S. Li , G. Li , K. B. Sharpless , P. Wu , J. Am. Chem. Soc. 2018, 140, 2919–2925. 29451783 10.1021/jacs.7b12788 PMC6192542

[62] P. Yamanushkin , K. Kaya , M. A. M. Feliciano , B. Gold , J. Org. Chem. 2022, 87, 3868–3973. 35143195 10.1021/acs.joc.1c03105 PMC9011340

[63] K. A. Andersen , M. R. Aronoff , N. A. McGrath , R. T. Raines , J. Am. Chem. Soc. 2015, 137, 2412–2415. 25658416 10.1021/ja5095815 PMC4372190

[64] E. D. Glendening, J. K. Badenhoop, A. E. Reed, J. E. Carpenter, J. A. Bohmann, C. M. Morales, P. Karafiloglou, C. R. Landis, F. Weinhold, NBO 7.0; Theoretical Chemistry Institute, University of Wisconsin-Madison: Madison, WI, **2018**.

[65a] D. Svatunek , N. Houszka , T. A. Hamlin , F. M. Bickelhaupt , H. Mikula , Chem. Eur. J. 2019, 25, 754–758; 30347481 10.1002/chem.201805215 PMC6391941

[65b] D. Svatunek , N. Houszka , T. A. Hamlin , F. M. Bickelhaupt , H. Mikula , Chem. Eur. J. 2022, 28, e202200414. 35285565 10.1002/chem.202200414 PMC9113636

[66a] N. A. McGrath , R. T. Raines , Chem. Sci. 2012, 3, 3237–3240; 23227302 10.1039/C2SC20806G PMC3513925

[66b] K. A. Mix , M. R. Aronoff , R. T. Raines , ACS Chem. Biol. 2016, 11, 3233–3244; 27739661 10.1021/acschembio.6b00810 PMC5161546

[66c] M. R. Aronoff , B. Gold , R. T. Raines , Org. Lett. 2016, 18, 1538–1541; 26981746 10.1021/acs.orglett.6b00278 PMC5141246

[66d] B. Gold , M. R. Aronoff , R. T. Raines , Org. Lett. 2016, 18, 4466–4469; 27599159 10.1021/acs.orglett.6b01938 PMC5148626

[66e] B. Gold , M. R. Aronoff , R. T. Raines , J. Org. Chem. 2016, 81, 5998–6006. 27332711 10.1021/acs.joc.6b00948 PMC5141247

[67a] J. M. J. M. Ravasco, J. A. S. Coelho, **2022**, DOI: 10.26434/chemrxiv-2022-tqh6t;

[67b] J. M. J. M. Ravasco , J. A. S. Coelho , J. Am. Chem. Soc. 2020, 142, 4235–4241. 32057243 10.1021/jacs.9b11948

[68a] Verloop, *Drug Design Vol III*, Academic Press, New York, NY, USA 1976;

[68b] L. Pauling , R. Corey , Proc. Natl. Acad. Sci. USA 1951, 37, 235–240. 14834145 10.1073/pnas.37.5.235 PMC1063348

[69] M. R. Karver , R. Weissleder , S. A. Hilderbrand , Angew. Chem. Int. Ed. 2012, 51, 920–922; 10.1002/anie.201104389 PMC3304098 22162316

[70] Y. Hu , J. M. Roberts , H. R. Kilgore , A. S. Mat Lani , R. T. Raines , J. M. Schomaker , J. Am. Chem. Soc. 2020, 142, 18826–18835. 33085477 10.1021/jacs.0c06725 PMC7891878

[71] K. Maegawa , H. Tanimoto , S. Onishi , T. Tomohiro , T. Morimoto , K. Kakiuchi , Org. Chem. Front. 2021, 8, 5793–5803.

[72] S. L. Scinto , D. A. Bilodeau , R. Hincapie , W. Lee , S. S. Nguyen , M. Xu , C. W. am Ende , M. G. Finn , K. Lang , Q. Lin , J. P. Pezacki , J. A. Prescher , M. S. Robillard , J. M. Fox , Nat. Rev. Methods. Primers. 2021, 1, 1–30. 10.1038/s43586-021-00028-z PMC8469592 34585143

